# Elucidation of the roles of *adhE1* and *adhE2* in the primary metabolism of *Clostridium acetobutylicum* by combining in-frame gene deletion and a quantitative system-scale approach

**DOI:** 10.1186/s13068-016-0507-0

**Published:** 2016-04-26

**Authors:** Minyeong Yoo, Christian Croux, Isabelle Meynial-Salles, Philippe Soucaille

**Affiliations:** INSA, UPS, INP, LISBP, Université de Toulouse, Toulouse, France; INRA, UMR792, Toulouse, France; CNRS, UMR5504, Toulouse, France; Metabolic Explorer, Biopôle Clermont-Limagne, Saint Beauzire, France

**Keywords:** AdhE, Butanol, *Clostridium acetobutylicum*, System-scale analysis

## Abstract

**Background:**

*Clostridium acetobutylicum* possesses two homologous *adhE* genes, *adhE1* and *adhE2*, which have been proposed to be responsible for butanol production in solventogenic and alcohologenic cultures, respectively. To investigate their contributions in detail, in-frame deletion mutants of each gene were constructed and subjected to quantitative transcriptomic (mRNA molecules/cell) and fluxomic analyses in acidogenic, solventogenic, and alcohologenic chemostat cultures.

**Results:**

Under solventogenesis, compared to the control strain, only Δ*adhE1* mutant exhibited significant changes showing decreased butanol production and transcriptional expression changes in numerous genes. In particular, *adhE2* was over expressed (126-fold); thus, AdhE2 can partially replace AdhE1 for butanol production (more than 30 % of the in vivo butanol flux) under solventogenesis. Under alcohologenesis, only Δ*adhE2* mutant exhibited striking changes in gene expression and metabolic fluxes, and butanol production was completely lost. Therefore, it was demonstrated that AdhE2 is essential for butanol production and thus metabolic fluxes were redirected toward butyrate formation. Under acidogenesis, metabolic fluxes were not significantly changed in both mutants except the complete loss of butanol formation in Δ*adhE2*, but numerous changes in gene expression were observed. Furthermore, most of the significantly up- or down-regulated genes under this condition showed the same pattern of change in both mutants.

**Conclusions:**

This quantitative system-scale analysis confirms the proposed roles of AdhE1 and AdhE2 in butanol formation that AdhE1 is the key enzyme under solventogenesis, whereas AdhE2 is the key enzyme for butanol formation under acidogenesis and alcohologenesis. Our study also highlights the metabolic flexibility of *C. acetobutylicum* to genetic alterations of its primary metabolism.

**Electronic supplementary material:**

The online version of this article (doi:10.1186/s13068-016-0507-0) contains supplementary material, which is available to authorized users.

## Background

*Clostridium acetobutylicum* is now considered as the model organism for the study of solventogenic Clostridia [1, 2]. The superiority of butanol over ethanol as an alternative biofuel has attracted research interest into *C. acetobutylicum* and other recombinant bacteria producing butanol as major products [3].

In phosphate-limited chemostat cultures, *C. acetobutylicum* can be maintained in three different stable metabolic states [4–8] without cellular differentiation [9]: acidogenic (producing acetate and butyrate) when grown at neutral pH with glucose; solventogenic (producing acetone, butanol, and ethanol) when grown at low pH with glucose; and alcohologenic (forming butanol and ethanol but not acetone) when grown at neutral pH under conditions of high NAD(P)H availability [5, 6, 10].

AdhE1 (CA_P0162 gene product, also referred to as Aad) has long been considered as an NADH-dependent bifunctional alcohol/aldehyde dehydrogenase responsible for alcohol formation in solventogenic *C. acetobutylicum* cultures [1, 2, 11]. Recently, however, AdhE1 was purified and shown to have lost most of its alcohol dehydrogenase activity despite its NADH-dependent aldehyde dehydrogenase activity [12].

Prior to the identification of *adhE2* (CA_P0035), the existence of alcohologenesis-specific gene(s) responsible for alcohol formation was predicted because (i) there was high NADH-dependent butanol dehydrogenase activity in alcohologenesis versus high NADPH-dependent butanol dehydrogenase activity in solventogenesis [5, 7] and (ii) previously identified genes related to butanol production (*bdhA*, *bdhB*, *adhE1*) were not induced in alcohologenic cultures [13]. The *adhE2* gene is the second aldehyde/alcohol dehydrogenase-encoding gene and is carried by the pSol1 megaplasmid, as is *adhE1* [14]. The two genes are not clustered, in contrast to the observations for *C. ljungdahlii* [15] and their expression patterns differ [9, 12]. *adhE1, ctfA,* and *ctfB* (CA_P0163 and CA_P0164) form the *sol* operon [1, 11]; *ctfA* and *ctfB* encode the CoA-transferase responsible for the first step of acetone formation, while the second step, catalyzed by acetoacetate decarboxylase, is encoded by *adc* (CA_P0165), located downstream of the *sol* operon. However, *adc* is transcribed under the control of its own promoter, which is oriented in the opposite direction of the *sol* operon [11].

In the three metabolic states, the contributions of the different enzymes responsible for the butyraldehyde dehydrogenase and butanol dehydrogenase activities to butanol flux have recently been characterized [12]. Under acidogenesis, the low butanol flux is catalyzed by AdhE2 (100 %) for butyraldehyde dehydrogenase activity, while BdhB and BdhA are responsible for butanol dehydrogenase activity. Under solventogenesis, AdhE1 (95 %; the other 5 % is contributed by AdhE2) is the key player responsible for butyraldehyde dehydrogenase activity, while BdhB, BdhA, and BdhC are responsible for butanol dehydrogenase activity. Under alcohologenesis, AdhE2 plays a major role in both butyraldehyde dehydrogenase (100 %) and butanol dehydrogenase activities. In the study of Cooksley et al. [16], *adhE1* and *adhE2* knockout mutants were (i) constructed using the ClosTron method [17] and (ii) phenotypically characterized in batch culture using Clostridium basal medium (CBMS) without pH adjustment. The *adhE1* knockout mutant obtained in their study exhibited low ethanol and no butanol formation along with scant acetone production; these findings were consistent with the polar effect of the intron on *ctfAB* transcription [16]. Using the *adhE2* knockout mutant, no alteration of solvent production was observed; however, the *adhE2* knockout mutant has not been evaluated under alcohologenic conditions, under which it is normally thought to play a major role [14].

The aim of this study was to perform clean individual in-frame deletions of *adhE1* and *adhE2* to characterize their roles in butanol formation in the three different metabolic states in more detail. Furthermore, to study the metabolic flexibility of *C. acetobutylicum* in response to each of these gene deletions, a complete fluxomic and quantitative transcriptomic analysis was also performed in the three conditions known for the wild-type strains: acidogenic, solventogenic, and alcohologenic states. The results presented here not only support our previous studies [12, 14] on the roles of AdhE1 and AdhE2 in butanol formation in different metabolic states but also highlight the metabolic flexibility of *C. acetobutylicum* to genetically alter its primary metabolism.

## Results and discussion

### Construction of *ΔadhE1* and *ΔadhE2* mutant strains

**Fig. 1:**
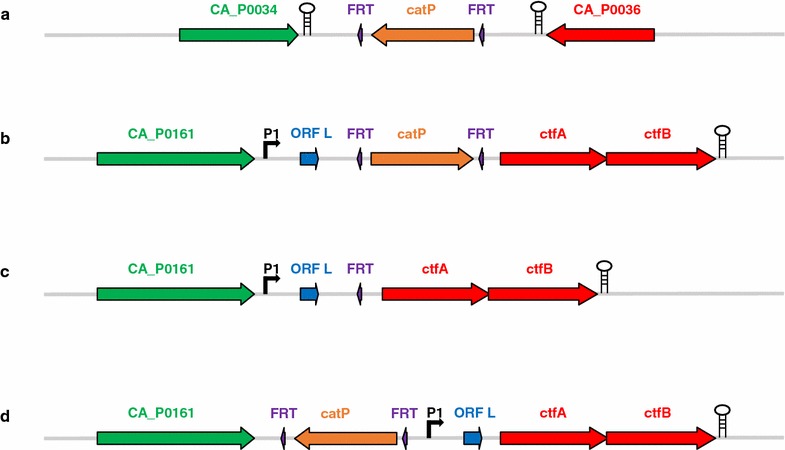
Construction of *ΔadhE1 and ΔadhE2*. The single construction of *ΔadhE2 and* three different constructions of *ΔadhE1* are described: *ΔCA_C1502ΔuppΔadhE2::catP* (**a**), *ΔCA_C1502ΔuppΔadhE1::catP* (**b**), *ΔCA_C1502ΔuppΔadhE1* (**c**), and *ΔCA_C1502ΔuppΔadhE1::catP*-*A1A4* (**d**). P1 indicating the promoter of the *sol* operon and ORF L were previously proposed by Fischer et al. [11]

**Fig. 2:**
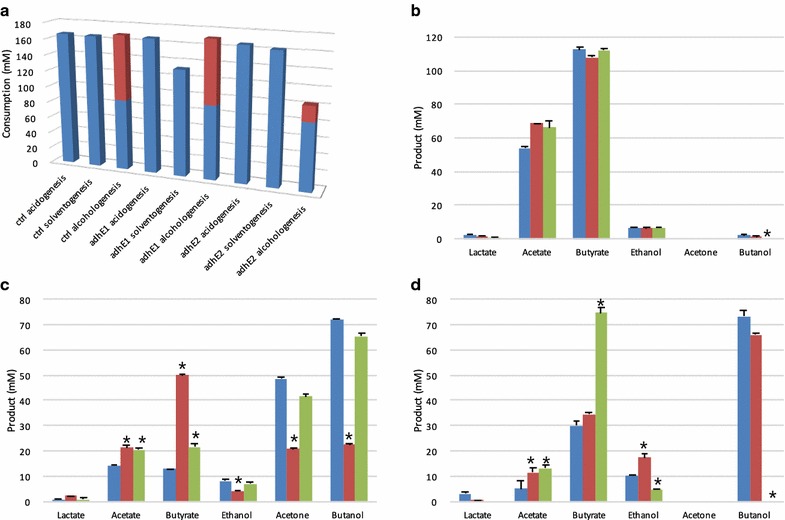
Substrates and products profile under three different conditions for the control, *ΔadhE1* and *ΔadhE2* strains. **a** Carbon source consumption: glucose (*blue*) and glycerol (*red*). Product profiles in acidogenesis (**b**), solventogenesis (**c**), and alcohologenesis (**d**). For (**b**), (**c**), and (**d**), each histogram indicates different strains: control (*blue*), *ΔadhE1* (*red*), and *ΔadhE2* (*green*). Each *error bar* indicates the SEM from the mean of duplicate samples. *The value is significantly different from the value of the control at the 1 % level based on the *P* value obtained from Student’s T-test

Construction of the *ΔadhE2* mutant was relatively straightforward, as *adhE2* is expressed in a monocistronic operon [14] (Fig. 1a). However, the position of *adhE1* as the first gene of the *sol* operon made the construction of *ΔadhE1* more complicated because the transcription of downstream *ctfAB* genes could be affected. Figure 1b–d shows different configurations of the *sol* operon promoter, *ctfAB* genes, and either *catP* cassette with two FRT (Flippase Recognition Target) sites or a single FRT site remaining after Flippase (Flp)-FRT recombination of the three different types of *ΔadhE1* mutants generated in this study. The first constructed *ΔadhE1* mutant, *ΔCA_C1502ΔuppΔadhE1::catP* (Fig. 1b), was unable to form acetone as predicted because a transcriptional terminator was included in the *catP* cassette, which is located upstream of *ctfAB* encoding the acetoacetyl coenzyme A:acetate/butyrate:coenzyme A transferase that is responsible for the first specific step of acetone formation [11]. However, after removing the *catP* cassette from *ΔCA_C1502ΔuppΔadhE1::catP*, acetone production was unexpectedly not recovered in *ΔCA_C1502ΔuppΔadhE1* (Fig. 1c). The presence of the megaplasmid pSOL1 was confirmed by the production of ethanol and butanol under alcohologenic conditions and was attributed to *adhE2* expression. By sequencing the pSOL1 region around the *adhE1* deletion, we confirmed that there was no mutation in the *sol* promoter, *ctfAB* and *adc* (encoding acetoacetate decarboxylase, which is responsible for the last step of acetone production). Based on these results, the possibility of unsuspected early transcriptional termination by the FRT site remaining after *catP* removal was deduced. To confirm the early termination of transcription by an FRT site and to eliminate this polar effect on acetone production, a new plasmid was constructed to position both of the FRT sites carried by the *catP* cassette upstream of the *sol* operon promoter and was used to construct the *ΔadhE1* mutant *ΔCA_C1502ΔuppΔadhE1::catP*-*A1A4* mutant (Fig. 1d). Consistent with our hypothesis, this last *ΔadhE1* mutant recovered acetone production (Fig. 2, Additional file 1: Fig. S3). To the best of our knowledge, the potential role of an FRT site as a transcriptional terminator was reported once in *Salmonella* [18] and twice in yeast [19, 20], although the FRT site is not generally recognized as possessing this additional activity. However, the high score of the FRT site hit from the “Dimers and Hairpin Loops analysis” in Vector NTI software (Invitrogen) and the detection of this activity upon deleting *adhE1* in *C. acetobutylicum* unambiguously demonstrate that the FRT site can function as a transcriptional terminator.

Hereafter, *C. acetobutylicum ΔCA_C1502ΔuppΔadhE1::catP*-*A1A4* (Fig. 1d) is referred to as *ΔadhE1* in all the chemostat culture experiments.

### Carbon and electron fluxes of *ΔadhE1* and *ΔadhE2* mutants under different physiological conditions

The *ΔadhE1* and *ΔadhE2* mutants were first evaluated under acidogenic conditions and compared to previously published data for the control strain [12]. All the strains behaved the same, and no significant changes in the metabolic fluxes were recorded (Additional file 1: Fig. S3), except that butanol production was completely abolished in the *ΔadhE2* mutant strain (Fig. 2, Additional file 1: Fig. S3).

The two mutant strains were then evaluated under solventogenic conditions and compared to previously published data for the control strain [12]. The control and *ΔadhE2* strains behaved the same, with no significant change in metabolic fluxes (Additional file 1: Fig. S3). However, the *ΔadhE1* mutant exhibited a completely different behavior. In the first phase, before the “pseudo steady state” was reached, this mutant exhibited considerable fluctuations in growth, glucose consumption, and metabolite profiles. Under “pseudo steady state conditions,” the butanol and acetone fluxes were stable, while the butyrate flux showed fluctuations between 2.2 and 2.9 mmol g^−1^ h^−1^. In *ΔadhE1*, the butanol, ethanol, and acetone fluxes decreased by 60, 49, and 46 %, respectively (Additional file 1: Fig. S3), compared to the control strain; thus, the acetone and ethanol fluxes were not reduced as greatly as the butanol fluxes. These results support the previously proposed [1, 11, 12, 14] key role of AdhE1 in butanol production under solventogenic conditions and demonstrate that an *adhE1* knockout strain with no polar effect on *ctfAB* transcription can still produce acetone. The level of *ctfAB* expression was 3-fold higher in the *adhE1* knockout compared to the control strain. This indicates that the lower flux of acetone production is the result of a control at the enzyme level due to a lower acetoacetyl-CoA concentration and/or higher acetyl-CoA/butyryl-CoA concentrations. The remaining ability of the *ΔadhE1* strain to produce butanol under solventogenesis is explained by the higher *adhE2* expression (~127-fold higher than the control strain, but only 25 mRNA molecules/cell) (Table 1, Additional file 2: Dataset S1). For the *ΔadhE1* mutant, the butyrate flux increased by 5-fold compared to the control strain (Additional file 1: Fig. S3), although neither *ptb*-*buk* (CA_C3076–CA_C3075) nor *buk2* (CA_C1660) experienced a significant transcriptional increase (Additional file 2: Dataset S1). Thus, flux is controlled at the enzyme level via an increase in the butyryl-CoA pool due to the lower flux in the butanol pathway. However, as the AdhE2 level in the mutant is the same as the AdhE1 level in the control (6.31 × 10^4^ versus 5.99 × 10^4^ protein molecules/cell), the lower flux of butanol production can be explained by (i) a lower catalytic efficiency of AdhE2 for butyryl-CoA and/or NADH or (ii) a lower intracellular pH under solventogenic conditions that would be less optimal for AdhE2 that is normally expressed under alcohologenic conditions at neutral pH. The second hypothesis can be eliminated as the previously measured intracellular pH [4, 21] in solventogenic and alcohologenic cells are relatively close (5.5 and 5.95, respectively) as the ΔpH is inverted (more acidic inside) under alcohologenic conditions [6]. Finally, as we will see below, the fact that ethanol flux is less affected than the butanol flux might be explained by the existence of an ethanol flux through the Pdc (pyruvate decarboxylase, encoded by CA_P0025) and bdhA/BdhB.

**Table 1:** Transcriptional changes of genes coding for the six key enzymes for alcohol production

Metabolic state/gene	Control	*ΔadhE1*	*ΔadhE2*
Acidogenesis			
*adhE1* (CA_P0162)	0.09 ± 0.01	0 ± 0	0.2 ± 0.01
*adhE2* (CA_P0035)	0.42 ± 0.02	2.31 ± 0.6	0 ± 0
*bdhA* (CA_C3299)	8.15 ± 0.32	4.33 ± 1.03	5.76 ± 0.2
*bdhB* (CA_C3298)	16.31 ± 0.45	5.13 ± 4.28	1.52 ± 0.11
*bdhC* (CA_C3392)	8.63 ± 0.94	7.55 ± 0.28	17.65 ± 0.44
*pdc* (CA_P0025)	5.6 ± 0.81	1.74 ± 0.1	3.23 ± 0.24
Solventogenesis			
*adhE1* (CA_P0162)	7.09 ± 0.73	0 ± 0	11.4 ± 4.71
*adhE2* (CA_P0035)	0.21 ± 0.02	26.6 ± 0.26	0 ± 0
*bdhA* (CA_C3299)	8.22 ± 1.33	4.62 ± 0.06	7.55 ± 0.75
*bdhB* (CA_C3298)	28.1 ± 5.07	34.78 ± 1.55	17.76 ± 2.83
*bdhC* (CA_C3392)	11.28 ± 1.68	12.52 ± 0.36	9.16 ± 0.67
*pdc* (CA_P0025)	5.17 ± 2.78	6.59 ± 0.3	6.23 ± 1.03
Alcohologenesis			
*adhE1* (CA_P0162)	0.13 ± 0.01	0 ± 0	0.18 ± 0.01
*adhE2* (CA_P0035)	68.6 ± 12.95	62.56 ± 7.58	0 ± 0
*bdhA* (CA_C3299)	6.08 ± 0.37	4.82 ± 0.13	7.39 ± 0.21
*bdhB* (CA_C3298)	14.33 ± 2.65	16.96 ± 0.25	15.16 ± 0.46
*bdhC* (CA_C3392)	10.73 ± 0.94	11.05 ± 0.25	8.95 ± 0.32
*pdc* (CA_P0025)	1.23 ± 0.51	0.83 ± 0.03	1.86 ± 0.07

The numbers of mRNA molecules per cell are shown as mean values ± SD from three biological replicates

**Fig. 3:**
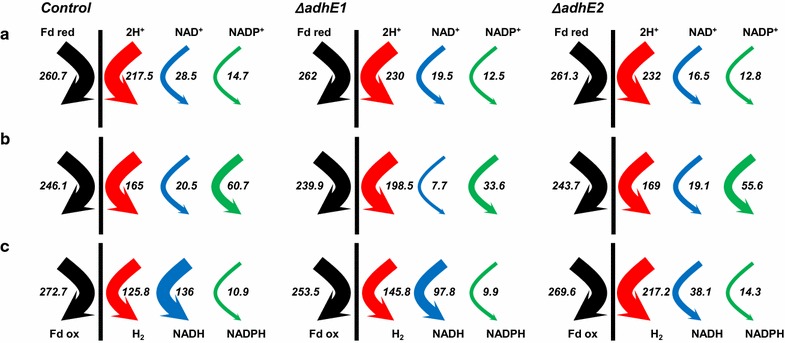
Electron flux map of the control, *ΔadhE1* and *ΔadhE2* strains in acidogenesis (**a**), solventogenesis (**b**), and alcohologenesis (**c**). The *arrows* for hydrogenase (*red*), ferredoxin-NAD + reductase (*blue*), and ferredoxin-NADP + (*green*) in vivo fluxes are presented. All values are normalized to the flux of the initial carbon source [millimoles per gram of dry cell weight (DCW) per hour]. Glucose flux is normalized to 100 for acidogenesis and solventogenesis, and the sum of glucose and half of the glycerol is normalized to 100 for alcohologenesis

The two mutant strains were also evaluated under alcohologenic conditions and compared to previously published data for the control strain [12]. The control and *ΔadhE1* strains behaved the same, with no significant changes in metabolic fluxes (Additional file 1: Fig. S3). However, the *ΔadhE2* mutant exhibited a completely different behavior; no flux toward butanol was detected, whereas fluxes toward butyrate became the primary fluxes, as opposed to butanol in the control strain (Additional file 1: Fig. S3). In addition, acetate levels increased by ~3-fold, and such changes were accompanied by changes in electron fluxes (Fig. 3), which are described in detail below. These phenomena were not observed by Cooksley et al. [16] with their *adhE2* knockout mutant, as they performed batch fermentation without promoting alcohologenic conditions. As *adhE1* was not expressed under the “alcohologenic conditions” of the *ΔadhE2* mutant, the physiological function of *adhE2* does not appear to be compensated by *adhE1* (Table 1). To verify that loss of the butanol-producing ability under alcohologenesis did not result from loss of the pSOL1 megaplasmid [22, 23] but rather from the deletion of *adhE2*, the culture was switched to solventogenic conditions before the experiment was ended; under solventogenic conditions, high butanol and acetone production fluxes were recovered (data not shown).

The butanol pathway was analyzed for three different conditions in the respective mutants (Additional file 1: Fig. S2) by calculating the contribution of each of the five enzymes potentially involved in each of the two steps to the fluxes (see methods for the calculation).

Under acidogenesis, *adhE1* was not expressed, and thus AdhE1 could not replace AdhE2 for the conversion of butyryl-CoA to butyraldehyde in the *ΔadhE2* mutant (Additional file 1: Fig. S2). This failure of AdhE1 to replace AdhE2 led to the absence of butanol production in the *ΔadhE1* mutant, which behaved the same as the control strain, leaving AdhE2 responsible for all the conversion. The *ΔadhE1* mutant behaved the same as the control strain with respect to the conversion of butyraldehyde to butanol under these conditions, and AdhE2 (45 % of the flux), BdhB (34 % of the flux), and BdhA (14 % of the flux) were the main contributors (Additional file 1: Fig. S2). The *ΔadhE2* mutant was not analyzed because it does not produce butanol.

Under solventogenesis, AdhE2 replaced AdhE1 for the conversion of butyryl-CoA to butyraldehyde in the *ΔadhE1* mutant, while in the *ΔadhE2* mutant, which behaved the same as the control strain, AdhE1 was responsible for all the conversion. The two main contributors to the conversion of butyraldehyde to butanol under these conditions were AdhE2 (67 % of the flux) and BdhB (30 % of the flux) in the *ΔadhE1* mutant, while in the *ΔadhE2* mutant, which behaved the same as the control strain, BdhB (75 % of the flux) and BdhA (16 % of the flux) were the main contributors (Additional file 1: Fig. S2).

Under alcohologenesis, *adhE1* was not expressed (Table 1, Additional file 2: Dataset S1), and thus, AdhE1 could not replace AdhE2 for the conversion of butyryl-CoA to butyraldehyde in the *ΔadhE2* mutant. This failure of AdhE1 to replace AdhE2 led to the absence of butanol production, while in the *ΔadhE1* mutant, which behaved the same as the control strain, AdhE2 was responsible for all the conversion. The *ΔadhE1* mutant behaved the same as the control strain with respect to the conversion of butyraldehyde to butanol under these conditions, and AdhE2 was the main contributor (Additional file 1: Fig. S2). The *ΔadhE2* mutant was not analyzed because it does not produce butanol.

Two possible routes are known for the conversion of pyruvate to acetaldehyde in *C. acetobutylicum*: (i) a two-step reaction by pyruvate:ferredoxin oxidoreductase (PFOR) and acetaldehyde dehydrogenase via acetyl-CoA production or (ii) a one-step reaction by pyruvate decarboxylase (Pdc, encoded by CA_P0025) [24]. In the wild-type strain, the former route is considered as the primary pathway [2, 25]. Under acidogenic and alcohologenic conditions of the *ΔadhE2* mutant, ethanol production was observed, but no butanol production was detected (Fig. 2, Additional file 1: Fig. S3). As previously reported [12], AdhE1 retains only aldehyde dehydrogenase activity, whereas AdhE2 possesses both aldehyde and alcohol dehydrogenases activities. Thus, the ethanol production of the *ΔadhE2* mutant suggests that the latter route is active. In other words, Pdc could be functional, and the ethanol dehydrogenase activity in acidogenesis could be due to BdhA, BdhB, or BdhC (Table 1). The same pathway might also be functional in solventogenesis and explains why in the *ΔadhE1* mutant the ethanol flux was less affected than the butanol flux.

Because the predominant use of reduced ferredoxin is for hydrogen production [12], no significant effects were observed under acidogenesis in both the *ΔadhE1* and *ΔadhE2* mutants with respect to electron flux (Fig. 3). In addition, solventogenesis of the *ΔadhE2* mutant exhibited similar flux levels to the control strain due to the small contribution of AdhE2 (5 % for butyraldehyde dehydrogenase function and 9 % for butanol dehydrogenase function) under these conditions in the control strain. However, under the same conditions as for *ΔadhE1*, both the fluxes for NADH, known as the partner of AdhE1 and AdhE2, and for NADPH, known as the partner of BdhA, BdhB, and BdhC, were reduced (by ~2.7-fold and 1.8-fold, respectively) due to decreased carbon fluxes toward alcohols (Fig. 3, Additional file 1: Fig. S3). The most striking changes were observed in the *ΔadhE2* mutant under alcohologenesis, in which the primary use of reduced ferredoxin was switched from NADH to hydrogen production. The absence of butanol formation resulted in a ~3.6-fold decreased flux toward NADH production and a 1.7-fold increased flux toward hydrogen production (Fig. 3).

### Common criteria used for quantitative transcriptomic analysis

To filter the data from only significant results, the same criteria used to compare the wild-type strain under different physiological conditions [12] were used to compare the mutants to the control strain. The first criterion was >4.0-fold higher expression or >4.0-fold lower expression in *ΔadhE1* or *ΔadhE2* than in the control strain under the same physiological condition, and the second criterion was >0.2 mRNA molecules per cell in at least one of the two strains being compared.

### Genes affected by *adhE1* or *adhE2* deletion under acidogenesis

**Fig. 4:**
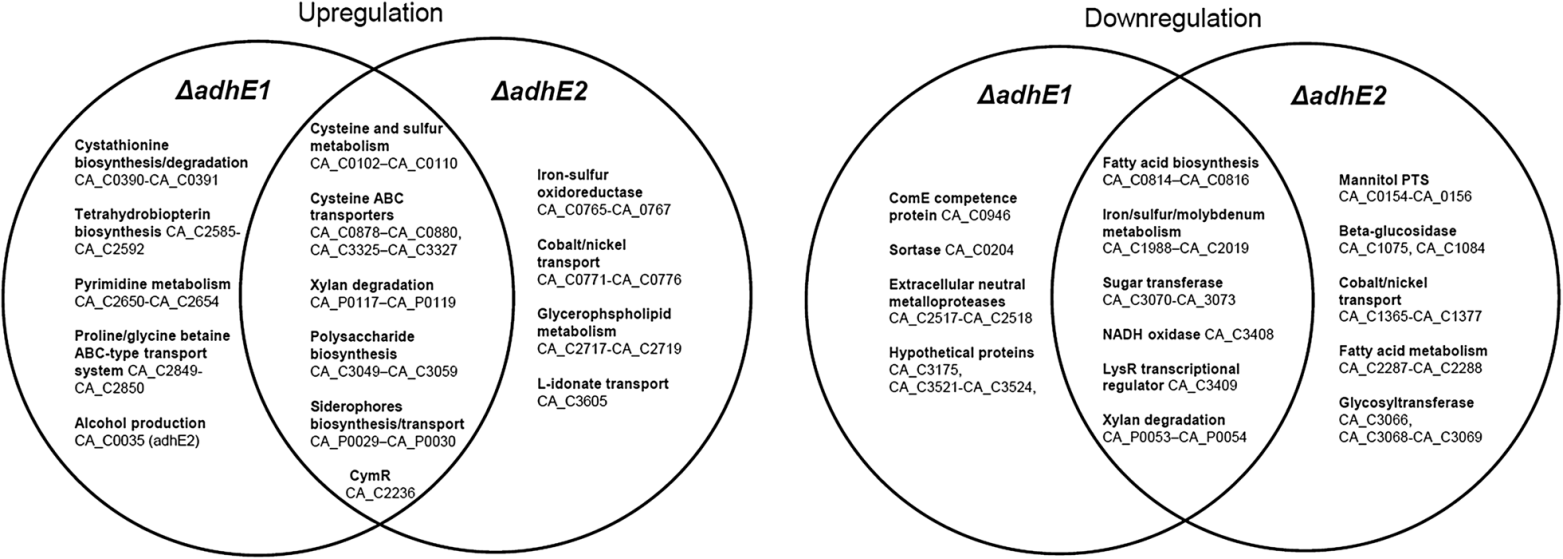
Venn diagrams of representative genes with involved pathways, which matched the significance criteria (>4-fold increase or decrease) in the *ΔadhE1* and *ΔadhE2* mutants. A complete list of each metabolic condition is provided in the Additional file 2

As alcohols are minor products under acidogenesis, the deletion of *adhE1* or *adhE2* did not significantly alter the metabolic flux map (Additional file 1: Fig. S3). However, a surprisingly large number of genes (100 genes increased in Δ*adhE1*, 108 genes decreased in *ΔadhE1*, 119 genes increased in Δ*adhE2*, 170 genes decreased in *ΔadhE2*) showed significant changes in mRNA molecules/cell in response to the deletion of each gene (Table 2). Furthermore, 50 genes (>4-fold increase) and 87 genes (>4-fold decrease) revealed the same patterns of change in both the *ΔadhE1* and *ΔadhE*2 mutants (Table 2). The primary metabolism-related genes that influence metabolic fluxes did not exhibit significant changes, whereas mostly subordinate metabolism-related genes were affected (Additional file 1: Table S2, Additional file 1: S3, and Fig. 4).

**Table 2:** Numbers of significantly changed genes by each gene deletion and genes exhibiting the same pattern of change for both deletions under three different metabolic states (the genes exhibiting the same pattern for both deletions under acidogenesis are listed in Table 3)

	Δ*adhE1*	Δ*adhE2*	Same pattern in Δ*adhE1* and Δ*adhE2*	Note^a^
Up-regulation under acidogenesis	100	119	50	Most CymR regulons are included
Down-regulation under acidogenesis	108	170	89	Most butanol response genes are included
Up-regulation under solventogenesis	55	22	0	
Down-regulation under solventogenesis	127	17	1	CA_C3612
Up-regulation under alcohologenesis	1	35	0	
Down-regulation under alcohologenesis	14	38	1	CA_C3274

^a^Representative features or locus number of the sole gene showing same pattern under certain condition are shown

Interestingly, a large portion (18 genes showed >a 4-fold increase, and 2 genes showed a >2.8-fold increase out of 30 genes proposed by Wang et al. [26]) of the cysteine metabolism regulator (CymR) regulon showed significantly increased expression in both mutants under acidogenesis (CymR regulons are indicated in Table 3). In particular, an operon involved in cysteine and sulfur metabolism (CA_C0102–CA_C0110) showed a >10-fold increase in both mutants. This operon was reported to respond to butyrate/butanol stresses and to be up-regulated under alcohologenesis in wild-type strains [12, 26, 27] and under solventogenesis in the *Δptb* mutant [28]. In addition, the expression of two putative cysteine ABC transporter operons belonging to the CymR regulon [26, 27], namely CA_C0878–CA_C0880 and CA_C3325–CA_C3327), was also up-regulated.

**Table 3:** Genes exhibiting the same pattern of change for both deletions under acidogenesis

Locus number	Function	*ΔadhE1*/Control strain	*ΔadhE2*/Control strain	Note^a^
Up-regulation				
CA_C0102	O-acetylhomoserine sulfhydrylase	28.70	20.49	CymR
CA_C0103	Adenylylsulfate kinase	32.55	22.06	CymR
CA_C0104	Adenylylsulfate reductase, subunit A	48.44	28.89	CymR
CA_C0105	Ferredoxin	30.78	21.84	CymR
CA_C0106	ABC-type probable sulfate transporter, periplasmic binding protein	26.09	14.54	CymR
CA_C0107	ABC-type sulfate transporter, ATPase component	22.86	13.03	CymR
CA_C0108	ABC-type probable sulfate transporter, permease protein	35.38	19.05	CymR
CA_C0109	Sulfate adenylate transferase, CysD subfamily	42.53	26.82	CymR
CA_C0110	GTPase, sulfate adenylate transferase subunit 1	54.78	42.48	CymR
CA_C0117	Chemotaxis protein cheY homolog	8.34	6.69	
CA_C0118	Chemotaxis protein cheA	11.00	8.24	
CA_C0119	Chemotaxis protein cheW	13.83	9.52	
CA_C0120	Membrane-associated methyl-accepting chemotaxis protein with HAMP domain	6.93	5.29	
CA_C0878	Amino acid ABC transporter permease component	5.61	4.04	CymR
CA_C0879	ABC-type polar amino acid transport system, ATPase component	8.29	5.60	CymR
CA_C0880	Periplasmic amino acid binding protein	9.50	6.50	CymR
CA_C0930	Cystathionine gamma-synthase	4.58	4.72	CymR
CA_C1392	Glutamine phosphoribosylpyrophosphate amidotransferase	4.20	4.47	
CA_C1394	Folate-dependent phosphoribosylglycinamide formyltransferase	4.11	4.57	
CA_C2072	Stage IV sporulation protein B, SpoIVB	**∞**	**∞**	
CA_C2235	Cysteine synthase/cystathionine beta-synthase, CysK	8.27	7.17	CymR
CA_C2236	Uncharacterized conserved protein of YjeB/RRF2 family	4.29	4.06	CymR encoding gene
CA_C2241	Cation transport P-type ATPase	7.92	7.62	
CA_C2242	Predicted transcriptional regulator, arsE family	5.01	5.22	
CA_C2521	Hypothetical protein, CF-41 family	4.33	5.70	
CA_C2533	Protein containing ChW-repeats	**∞**	**∞**	
CA_C2816	Hypothetical protein, CF-17 family	6.00	11.20	
CA_C3049	Glycosyltransferase	4.79	7.42	
CA_C3050	AMSJ/WSAK-related protein, possibly involved in exopolysaccharide biosynthesis	4.70	8.25	
CA_C3051	Glycosyltransferase	5.16	9.60	
CA_C3052	Glycosyltransferase	5.59	9.91	
CA_C3053	Histidinol phosphatase-related enzyme	7.03	10.94	
CA_C3054	Phosphoheptose isomerase	6.69	11.37	
CA_C3055	Sugar kinase	5.90	10.87	
CA_C3056	Nucleoside-diphosphate-sugar pyrophosphorylase	6.37	11.28	
CA_C3057	Glycosyltransferase	12.36	11.92	
CA_C3058	Mannose-1-phosphate guanylyltransferase	9.94	11.59	
CA_C3059	Sugar transferases	13.47	12.63	
CA_C3325	Periplasmic amino acid binding protein	18.24	10.68	CymR
CA_C3326	Amino acid ABC-type transporter, permease component	19.82	11.79	CymR
CA_C3327	Amino acid ABC-type transporter, ATPase component	28.33	16.73	CymR
CA_C3461	Hypothetical protein	4.52	16.79	
CA_C3556	Probable S-layer protein;	4.18	10.41	
CA_C3636	Oligopeptide ABC transporter, ATPase component	4.23	4.68	
CA_P0029	Permease MDR-related	**∞**	**∞**	
CA_P0030	Isochorismatase	385.91	81.89	
CA_P0031	Transcriptional activator HLYU, HTH of ArsR family	46.17	10.93	
CA_P0117	Possible beta-xylosidase diverged, family 5/39 of glycosyl hydrolases and alpha-amylase C (Greek key) C-terminal domain	56.53	4.94	
CA_P0118	Possible xylan degradation enzyme (glycosyl hydrolase family 30-like domain and Ricin B-like domain)	54.97	5.22	
CA_P0119	Possible xylan degradation enzyme (glycosyl hydrolase family 30-like domain and Ricin B-like domain)	46.44	4.23	
Down-regulation				
CA_C0078	Accessory gene regulator protein B	0.04	0.00	
CA_C0079	Hypothetical protein	0.00	0.00	
CA_C0082	Predicted membrane protein	0.02	0.00	
CA_C0310	Regulators of stationary/sporulation gene expression, abrB B.subtilis ortholog	0.15	0.23	
CA_C0381	Methyl-accepting chemotaxis protein	0.18	0.13	
CA_C0437	Sensory transduction histidine kinase	0.15	0.23	
CA_C0537	Acetylxylan esterase, acyl-CoA esterase or GDSL lipase family, strong similarity to C-terminal region of endoglucanase E precursor	0.15	0.10	
CA_C0542	Methyl-accepting chemotaxis protein	0.21	0.08	
CA_C0658	Fe-S oxidoreductase	0.24	0.00	
CA_C0660	Hypothetical protein, CF-26 family	0.17	0.08	BuOH
CA_C0814	3-oxoacyl-[acyl-carrier-protein] synthase III	0.11	0.02	BuOH
CA_C0815	Methyl-accepting chemotaxis protein	0.13	0.04	BuOH
CA_C0816	Lipase-esterase-related protein	0.17	0.04	BuOH
CA_C1010	Predicted phosphohydrolase, Icc family	0.21	0.04	BuOH
CA_C1022	Thioesterase II of alpha/beta hydrolase superfamily	0.22	0.11	
CA_C1078	Predicted phosphohydrolase, Icc family	0.17	0.04	BuOH
CA_C1079	Uncharacterized protein, related to enterotoxins of other Clostridiales	0.15	0.05	
CA_C1080	Uncharacterized protein, probably surface-located	0.11	0.01	
CA_C1081	Uncharacterized protein, probably surface-located	0.13	0.01	
CA_C1532	Protein containing ChW-repeats	0.22	0.08	
CA_C1766	Predicted sigma factor	0.19	0.00	
CA_C1775	Predicted membrane protein	0.16	0.05	
CA_C1868	Uncharacterized secreted protein, homolog YXKC Bacillus subtilis	0.22	0.18	
CA_C1989	ABC-type iron (III) transport system, ATPase component	0.18	0.11	BuOH
CA_C1991	Uncharacterized protein, YIIM family	0.23	0.10	BuOH
CA_C1993	Molybdenum cofactor biosynthesis enzyme MoaA, Fe-S oxidoreductase	0.23	0.18	BuOH
CA_C1994	Molybdopterin biosynthesis enzyme, MoaB	0.22	0.11	BuOH
CA_C1996	Hypothetical protein	0.19	0.08	BuOH
CA_C1997	Predicted glycosyltransferase	0.19	0.07	BuOH
CA_C1998	ABC-type transport system, ATPase component	0.19	0.07	BuOH
CA_C1999	Uncharacterized protein related to hypothetical protein Cj1507c from Campylobacter jejuni	0.20	0.07	BuOH
CA_C2000	Indolepyruvate ferredoxin oxidoreductase, subunit beta	0.19	0.06	BuOH
CA_C2001	Indolepyruvate ferredoxin oxidoreductase, subunit alpha	0.13	0.04	BuOH
CA_C2002	Predicted iron-sulfur flavoprotein	0.16	0.05	BuOH
CA_C2003	Predicted permease	0.16	0.08	BuOH
CA_C2004	Siderophore/Surfactin synthetase-related protein	0.10	0.04	BuOH
CA_C2005	Siderophore/Surfactin synthetase-related protein	0.12	0.05	BuOH
CA_C2006	Enzyme of siderophore/surfactin biosynthesis	0.15	0.07	BuOH
CA_C2007	Predicted glycosyltransferase	0.09	0.03	BuOH
CA_C2008	3-oxoacyl-(acyl-carrier-protein) synthase	0.11	0.04	BuOH
CA_C2009	3-Hydroxyacyl-CoA dehydrogenase	0.10	0.03	BuOH
CA_C2010	Predicted Fe-S oxidoreductase	0.09	0.03	BuOH
CA_C2011	Possible 3-oxoacyl-[acyl-carrier-protein] synthase III	0.12	0.03	BuOH
CA_C2012	Enoyl-CoA hydratase	0.12	0.04	BuOH
CA_C2013	Hypothetical protein	0.12	0.03	BuOH
CA_C2014	Predicted esterase	0.12	0.02	BuOH
CA_C2015	Hypothetical protein	0.15	0.04	BuOH
CA_C2016	Enoyl-CoA hydratase	0.12	0.02	BuOH
CA_C2017	Acyl carrier protein	0.15	0.03	BuOH
CA_C2018	Aldehyde:ferredoxin oxidoreductase	0.12	0.03	BuOH
CA_C2019	Malonyl CoA-acyl carrier protein transacylase	0.12	0.02	BuOH
CA_C2020	Molybdopterin biosynthesis enzyme, MoeA, fused to molybdopterin-binding domain	0.20	0.07	
CA_C2021	Molybdopterin biosynthesis enzyme, MoeA (short form)	0.24	0.06	
CA_C2023	Membrane protein, related to copy number protein COP from Clostridium perfringens plasmid pIP404 (GI:116,928)	0.22	0.12	
CA_C2026	Predicted flavodoxin	0.20	0.09	
CA_C2107	Contains cell adhesion domain	0.20	0.08	
CA_C2293	Hypothetical secreted protein	0.13	0.10	
CA_C2581	6-pyruvoyl-tetrahydropterin synthase-related domain; conserved membrane protein	0.24	0.11	BuOH
CA_C2663	Protein containing cell wall hydrolase domain	0.23	0.09	
CA_C2695	Diverged Metallo-dependent hydrolase(Zn) of DD-Peptidase family; peptodoglycan-binding domain	0.17	0.12	BuOH
CA_C2807	Endo-1,3(4)-beta-glucanase family 16	0.21	0.02	
CA_C2808	Beta-lactamase class C domain (PBPX family) containing protein	0.20	0.04	
CA_C2809	Predicted HD superfamily hydrolase	0.14	0.02	
CA_C2810	Possible glucoamylase (diverged), 15 family	0.14	0.01	
CA_C2944	N-terminal domain intergin-like repeats and c-terminal- cell wall-associated hydrolase domain	0.23	0.06	BuOH
CA_C3070	Glycosyltransferase	0.21	0.04	
CA_C3071	Glycosyltransferase	0.21	0.03	
CA_C3072	Mannose-1-phosphate guanylyltransferase	0.18	0.02	
CA_C3073	Sugar transferase involved in lipopolysaccharide synthesis	0.23	0.03	
CA_C3085	TPR-repeat-containing protein; Cell adhesion domain	0.25	0.12	
CA_C3086	Protein containing cell adhesion domain	0.20	0.11	
CA_C3251	Sensory transduction protein containing HD_GYP domain	0.20	0.11	
CA_C3264	Uncharacterized conserved protein, YTFJ B.subtilis ortholog	0.19	0.15	BuOH
CA_C3265	Predicted membrane protein	0.08	0.11	
CA_C3266	Hypothetical protein	0.07	0.07	
CA_C3267	Specialized sigma subunit of RNA polymerase	0.15	0.16	
CA_C3280	Possible surface protein, responsible for cell interaction; contains cell adhesion domain and ChW-repeats	0.23	0.14	
CA_C3408	NADH oxidase (two distinct flavin oxidoreductase domains)	0.03	0.02	
CA_C3409	Transcriptional regulators, LysR family	0.02	0.01	
CA_C3412	Predicted protein-S-isoprenylcysteine methyltransferase	0.22	0.06	
CA_C3422	Sugar:proton symporter (possible xylulose)	0.05	0.03	
CA_C3423	Acetyltransferase (ribosomal protein N-acetylase subfamily)	0.04	0.03	
CA_C3612	Hypothetical protein	0.18	0.00	BuOH
CA_P0053	Xylanase, glycosyl hydrolase family 10	0.24	0.09	BuOH
CA_P0054	Xylanase/chitin deacetylase family enzyme	0.24	0.07	BuOH
CA_P0057	Putative glycoprotein or S-layer protein	0.21	0.13	BuOH
CA_P0135	Oxidoreductase	0.25	0.21	
CA_P0136	AstB/chuR/nirj-related protein	0.25	0.23	
CA_P0174	Membrane protein	0.25	0.14	

^a^CymR indicates CymR regulon, BuOH indicates the genes to be down-regulated by butanol stress in an acidogenic chemostat in the study by Schwarz et al. [30]

A long gene cluster linked to iron/sulfur/molybdenum metabolism (CA_C1988–CA_C2019) exhibited significantly decreased expression (except for CA_C1988, CA_C1990, CA_C1992 and CA_C1995, for which some values were below the significance criterion of 4-fold but were higher than 3-fold) (Table 3, Additional file 2: Dataset S1). A part of this cluster, CA_C1988–CA_C1996, was previously reported to be down-regulated under oxygen-exposed conditions [29]. Moreover, this cluster was shown by Schwarz et al. [30] to be repressed by butanol stress in an acidogenic chemostat.

### Transcriptional changes due to *adhE1* or *adhE2* deletion under solventogenesis

Under solventogenesis, a drastic change in fluxes was observed in the *ΔadhE1* mutant, while the fluxes remained unchanged in the *ΔadhE2* mutant; additionally, as expected, more genes showed significant changes in *ΔadhE1* than in *ΔadhE2* (Table 2, Additional file 1: Table S4, Additional file 1: S5). Specifically, in *ΔadhE1,* 55 genes were up-regulated, and 127 genes were down-regulated (Table 2). In *ΔadhE2*, 22 genes were up-regulated, and 17 genes were down-regulated (Table 2). In contrast to the observations previously made under acidogenesis, no gene was significantly increased in both the *ΔadhE1* and *ΔadhE*2 mutants, and only 1 gene (CA_C3612, encoding a hypothetical protein) was significantly decreased in both mutants.

In *ΔadhE1*, the CA_C0102–CA_C0110 operon which was shown to be up-regulated in acidogenesis and belongs to the CymR regulon, was also up-regulated by >18-fold under solventogenesis (Additional file 1: Table S4). However, the up-regulation of this operon (under alcohologenesis in the control strain, acidogenesis and solventogenesis in *ΔadhE1*, or acidogenesis in *ΔadhE2*) did not have striking shared features with the main product profile.

Interestingly, expression of the *natAB* operon (CA_C3551–CA_C3550) (>10-fold), encoding a potential Na^+^-ABC transporter, and the *kdp* gene cluster (CA_C3678–CA_C3682), encoding a potential K^+^ transporter (>20-fold), was highly up-regulated under solventogenesis (Additional file 1: Table S4, Additional file 2: Dataset S1) in *ΔadhE1*. The *natAB* operon and the *kdp* gene cluster have previously been reported to be up-regulated by both acetate and butyrate stress [27]. As the ability of the *ΔadhE1* mutant to produce butanol was highly affected and as butyrate and acetate were the primary fermentation products (Fig. 2), this strain struggled to survive under acidic conditions (i.e., under the pH of 4.4 for solventogenesis); consequently, genes involved in ion transport were up-regulated.

The operon CA_P0029–CA_P0030, which potentially encodes a transporter and an isochorismatase, was up-regulated under acidogenesis in both mutants as well as under solventogenesis in *ΔadhE2* (>20-fold) (Table 2, Additional file 1: Table S5). Two neighboring genes, CA_C3604 (*ilvD*), encoding dihydroxyacid dehydratase linked to valine/leucine/isoleucine biosynthesis, and CA_C3605 (*gntP*), encoding high affinity gluconate/L-idonate permease, exhibited striking increases (>120-fold) (Additional file 1: Table S5) in *ΔadhE2*.

As described above, the solventogenic culture of *ΔadhE1* has a lower glucose consumption rate than the control strain (Fig. 2) and consequently more glucose remained unconsumed in the medium. Accordingly, numerous genes related to sugar metabolism were down-regulated under this metabolic state. For instance, all the structural genes on the mannitol phosphotransferase system (PTS)-related operon *mtlARFD* (CA_C0154–CA_C0157) and the mannose PTS-related operon (CA_P0066–CA_P0068) were decreased by >10-fold (Additional file 1: Table S4).

Interestingly, one of two operons encoding a quorum-sensing system and putatively involved in sporulation, CA_C0078–CA_C0079 (*agrBD*) [31], was strongly down-regulated (infinity-fold for CA_C0078 and 667-fold for CA_C0078) in *ΔadhE2* relative to the control strain (Additional file 1: Table S5). However, the other operon, CA_C0080–CA_C0081 (*agrCA*), did not significantly change (<3-fold decreases) (Additional file 2: Dataset S1). Quantitatively, less than 1 *agrCA* mRNA molecule was found per cell, whereas more than 1 *agrBD* mRNA molecule was found per cell under all conditions in the control strain [12]. These different expression levels are not surprising because *agrBD* and *agrCA* are independently transcribed [31–33]. In addition, *agrBD* was repressed under all conditions in *ΔadhE2*, although the sporulation of this mutant was not affected (Additional file 2: Dataset S1).

### Transcriptional changes due to *adhE1* or *adhE2* deletion under alcohologenesis

Under alcohologenesis, a drastic change in fluxes was observed in the *ΔadhE2* mutant, while in the *ΔadhE1* mutant, the fluxes remained unchanged. As expected, more genes showed significant changes in the *ΔadhE2* mutant than in the *ΔadhE1* mutant (Table 2). Specifically, in *ΔadhE1*, only 1 gene was up-regulated (*agrB),* and 14 genes were down-regulated, while in *ΔadhE2,* 35 genes were up-regulated, and 38 genes were down-regulated.

The most dynamic changes in the *ΔadhE2* mutant were observed in CA_C3604 (*ilvD*, 297-fold) and CA_C3605 (*gntP*, 301-fold) (Additional file 1: Table S7). As mentioned previously, these genes were highly up-regulated (>84-fold) under all the conditions in the *ΔadhE2* mutant (Additional file 2: Dataset S1). Interestingly, two genes located immediately downstream of *adhE2,* CA_P0036, which encodes a cytosolic protein of unknown function, and CA_P0037, which encodes a potential transcriptional regulator, exhibited a ~ 9-fold increase under alcohologenesis (Additional file 1: Table S7) in *ΔadhE2.*

A sucrose metabolism operon comprising s*crAKB* (CA_C0423–CA_C0425), encoding a PTS IIBCA domain on a single gene, fructokinase and sucrose-6-P hydrolase [35, 36], was strikingly down-regulated (>47-fold) (Additional file 1: Table S6). Moreover, the gene immediately upstream, *scrT* (CA_C0422) (encoding a putative transcriptional antiterminator), and the gene downstream, CA_C0426, encoding a putative AraC-type of regulator, were also decreased, by 9.3-fold and 8-fold, respectively (Additional file 1: Table S6). The similar expression patterns of CA_C0422, CA_C0426, and *scrAKB* support the hypotheses of previous studies regarding their roles in regulating *scrAKB* [35, 36].

As expected based on the reduced consumption of glycerol (approximately one-fourth of the control strain) (Fig. 2) in *ΔadhE2*, the gene cluster for glycerol transport and utilization (CA_C1319-CA_C1322) was down-regulated (>4.3-fold) under these conditions (Additional file 1: Table S7).

Most arginine biosynthesis-related genes known to respond negatively to butanol and butyrate stress [26] (i.e., CA_C0316 (*argF/I*), CA_C0973–CA_C0974 (*argGH*), CA_C2389–CA_C2388 (*argBD*), CA_C2390–CA_C2391 (*argCJ*), CA_C2644 (*carB*), and CA_C2645 (*carA*)) were significantly down-regulated (>4-fold decrease) (Additional file 1: Table S7) in *ΔadhE2*. As “alcohologenic cultures” of *ΔadhE2* produced 70 mM of butyrate and no butanol (Fig. 2), this down-regulation is consistent with the high butyrate stress (50 mM) response [26].

CA_C3486, which encodes a multimeric flavodoxin, was decreased by 4.4-fold in *ΔadhE2* (Additional file 1: Table S7), resulting in a loss of butanol production under alcohologenesis. This finding is consistent with the proposed hypothesis [12] that under alcohologenesis, the gene product of CA_C3486 may function as a redox partner between the hydrogenase and ferredoxin-NAD^+^ reductase and may participate in the redistribution of electron fluxes in favor of butanol formation.

## Conclusions

The results presented here support the hypothesis of the roles of AdhE1 and AdhE2 in butanol formation, namely that AdhE1 is the key enzyme for butanol formation in solventogenesis and that AdhE2 is the key enzyme for butanol formation in alcohologenesis. Furthermore, this study also demonstrates the metabolic flexibility of *C. acetobutylicum* in response to genetic alteration of its primary metabolism.

## Methods

### Bacterial strains and plasmid construction

All *C. acetobutylicum* strains used in this study and in the control study were constructed from the *C. acetobutylicum* ATCC 824 Δ*CA_C1502* Δ*upp* mutant strain, which was constructed for rapid gene knockout and gene knockin [38]. Detailed procedures, including all strains and primers used, are described in the online supporting information (Supplementary experimental procedures).

### Culture conditions

All batch cultures were performed under strict anaerobic conditions in synthetic medium (MS), as previously described [4]. *C. acetobutylicum* was stored in spore form at −20 °C after sporulation in MS medium. Heat shock was performed for spore germination by immersing the 30- or 60-mL bottle into a water bath at 80 °C for 15 min.

All the phosphate-limited continuous cultivations were performed as previously described by Vasconcelos et al. [4] and Girbal et al. [21] like in the control strain study [12]. The chemostat was fed a constant total of 995 mM of carbon and maintained at a dilution rate of 0.05 h^−1^. The maintained pH of the bioreactor and the supplied carbon sources of each metabolic state were as follows: for acidogenesis, pH 6.3, with 995 mM of carbon from glucose; for solventogenesis, pH 4.4, with 995 mM of carbon from glucose; and for alcohologenesis, pH 6.3, with 498 mM of carbon from glucose and 498 mM of carbon from glycerol.

### RNA extraction and microarray

Total RNA isolation and microarray experiments were performed as previously described [12]. Briefly, 3 mL of chemostat cultures was sampled, immediately frozen in liquid nitrogen and ground with 2-mercaptoethanol. RNA was extracted by using an RNeasy Midi kit (Qiagen, Courtaboeuf, France) and RNase-Free DNase Set (Qiagen) per the manufacturer’s protocol. The RNA quantity and integrity were monitored using an Agilent 2100 Bioanalyzer (Agilent Technologies, Massy, France) and a NanoDrop ND-1000 spectrophotometer (Labtech France, Paris, France) at 260 and 280 nm. All microarray procedures were performed per the manufacturer’s protocol (Agilent One-Color Microarray-Based Exon Analysis).

### Analytical methods

The optical density at 620 nm (OD620 nm) was monitored and used to calculate the biomass concentration with the correlation factor between dry cell weight and OD620 nm (path length 1 cm) of 0.28, which was experimentally determined from continuous cultures and was used in a control strain study [12]. The glucose, glycerol, acetate, butyrate, lactate, pyruvate, acetoin, acetone, ethanol, and butanol concentrations were determined using high-performance liquid chromatography (HPLC), as described by Dusséaux et al. [39]. The concentration of the eluent H_2_SO_4_ was changed to 0.5 mM, as this concentration was required to optimize the mobile phase for the control strain study [12].

### Calculation of the cytosolic proteins concentration (protein molecules per cell)

In a previously published work [12], we quantified the amount of (i) mRNA molecules per cell for all genes and (ii) protein molecules per cell (for approximately 700 cytosolic proteins) for steady-state chemostat cultures (at a specific growth rate of 0.05 h^−1^) of *C. acetobutylicum* under different physiological conditions. For 96 % of the cytosolic proteins that could be quantified, a linear relationship was obtained, with an R^2^ > 0.9, when the numbers of protein molecules per cell were plotted against the numbers of mRNA molecules per cell. This result indicated that for steady-state continuous cultures run at the same specific growth rate and with the same total amount of carbon supplied, the rate of protein turnover is proportional to the mRNA content for 96 % of the genes. As the mutants were cultivated in chemostat culture at the same growth rate (0.05 h^−1^), we used the absolute protein synthesis rates previously calculated [12] for each of the 700 genes to calculate the amount of protein molecule per cell for each of these 700 genes in the different mutants. (Additional file 2: Dataset S1).

### Calculation of the contribution of different enzymes on the butanol flux

The contribution of the 5 proteins potentially involved in the butanol pathway, namely AdhE1, AdhE2, BdhA, BdhB, and BdhC, was made as previously described [12] by assuming that all five enzymes function at their Vmax and using the calculated amount of each protein per cell (Additional file 2: Dataset S1).

## Additional files

10.1186/s13068-016-0507-0 Supplementary experimental procedures and results.
10.1186/s13068-016-0507-0 Dataset S1. Transcriptomic data of the total open reading frames (ORFs).

## Abbreviations

Flp: flippase
FRT: flippase recognition target
catP: chloramphenicol acetyltransferase
