# Supplementary material for: Elucidation of the roles of adhE1 and adhE2 in the primary metabolism of Clostridium acetobutylicum by combining in-frame gene deletion and a quantitative system-scale approach
Source: Biotechnol Biofuels. 2016 Apr 26;9:92. doi: 10.1186/s13068-016-0507-0 (PMC4845359; doi:10.1186/s13068-016-0507-0)
Supplement: Supplementary file 1 — 10.1186/s13068-016-0507-0 Supplementary experimental procedures and results. [file 13068_2016_507_MOESM1_ESM.doc]

Elucidation of the roles of *adhE1* and *adhE2* in the primary metabolism of *Clostridium acetobutylicum* by combining in-frame gene deletion and a quantitative system-scale approach

Minyeong Yoo, Christian Croux, Isabelle Meynial-Salles, and Philippe Soucaille

**Supporting Information**

**Experimental procedures**

***Plasmid constructions***

All primers used in this study are listed in Table S1. The allelic exchange method described by Croux *et al*. was used for deletion of target genes.

***Construction of pSOS95-upp***

The pSOS95-upp plasmid was constructed from the pSOS95-upp-DldhA-catP*a plasmid initially designed for the deletion of the *ldhA* gene. The pSOS95-upp-DldhA-catP* plasmid was constructed as follows. First two DNA fragments surrounding the *ldhA* gene (CA_C0267) were amplified by PCR using *C. acetobutylicum* ATCC824 total gDNA as template and two pairs of oligonucleotides as primers with the Phusion DNA Polymerase. Using pairs of primers ldhA-1/ldhA-2 and ldhA-3/ldhA-4, two 1 kb DNA fragments were obtained respectively. Both primers ldhA-1 and ldhA-4 introduce a *BamHI* site while primers ldhA-2 and ldhA-3 have complementary 5’ extended sequences which introduce a *StuI* site. DNA fragments ldhA-1/ ldhA-2 and ldhA-3/ ldhA-4 were joined in a PCR fusion with primers ldhA-1 and ldhA-4 and the resulting fragment was cloned into the pSC-B vector (Agilent) to generate pSCB-DldhA.

The Pptb-catP* cassette containing the modified antibiotic resistance catP gene under the control of the *C. acetobutylicum* phosphotransbutyrylase (ptb) promoter and flanked by two FRT sequences was obtained by PCR amplification with the Phusion DNA Polymerase using pSOS94-catP* plasmid as template and FRT-CM-F1 and FRT-CM-F2 oligonucleotides as primers. This 1.2 kb fragment was then cloned into the pSC-B vector to give the pSCB-FRT-catP* plasmid.

The 1kb *SmaI*/*HindIII* fragment from pSCB-FRT-catP* blunt-ended by klenow treatment was then cloned into the unique *StuI* site of the pSCB-DldhA to give the resulting pSCB-DldhA-FRT-catP*plasmid.

The 3.8 kb *BspHI*/*ClaI* blunt-ended by klenow treatment fragment from pSCB-DldhA-FRT-catP* was finally cloned into the *PstI*/E*coRI* digested and blunt ended pSOS95 backbone to give the pSOS95-DldhA-FRT-catP* plasmid.

The *upp* gene (CA_C2879) was amplified by PCR using *C. acetobutylicum ATCC824* total DNA as template and oligonucleotides Rep-upp-F and Rep-upp-F as primers with the Phusion DNA Polymerase. After *PvuII* digestion, the 0.7 kb resulting fragment was cloned into the *ClaI* digested and Klenow treated pSOS95-DldhA-FRT-catP* plasmid. The clones showing an insertion of the *upp* gene in the same orientation of that of the MLSR gene, *i.e.* resulting in the formation of an artificial bicistronic operon, were selected, and the final plasmid was named pSOS95-upp-DldhA-catP*.

After a *BamHI* digestion of the pSOS95-upp-DldhA-catP* plasmid to remove the specific region for *ldhA* deletion, and a self-ligation of the large fragment (5.6 kb), the pSOS95-upp plasmid was obtained, that can be subsequently used as a parental vector for the cloning into the unique *BamHI* site of others deletions-replacement cassettes (see deletion of *adhE1* and deletion of *adhE2*)

***Construction of pSOS95-upp-flp***

The 0.7 kb *PvuII* fragment containing the *upp* gene (see *ldhA* deletion) was cloned into the *ClaI* digested and blunt-ended (T4 DNA Polymerase) pSOS-catP* plasmid . The clones showing an insertion of the *upp* gene in the same orientation of that of the MLSR gene, *ie* resulting in the formation of an artificial bicistronic operon were selected, and the final plasmid named pSOS95-catP*-upp

The 1.6 kb *SalI* fragment from pCLF1 (WO2008040387) carrying the *Flp* gene under the control of the *C. acetobutylicum* thiolase (*thlA*) promoter was then introduced into the *SalI* digested and dephosphorylated pSOS95-catP*-upp backbone (catP* removing) to give the pSOS95-upp-Flp plasmid, designed for the removing of catP* antibiotic resistance cassette based on Flp-FRT recombination system.

***Deletion of adhE1***

Two DNA fragments surrounding the *adhE1* gene (CA_P0162) were amplified by PCR using *C. acetobutylicum* ATCC824 total DNA as template and two pairs of oligonucleotides as primers with the Phusion DNA Polymerase. Using pairs of primers adhE1-1/adhE1-2 and adhE1-3/adhE1-4, 1.1 kb and 1.2 kb DNA fragments were obtained respectively. Both primers adhE1-1 and adhE1-4 introduce a *BglII* site while primers adhE1-2 and adhE1-3 have complementary 5’ extended sequences which introduce a *StuI* site. adhE1-2 was designed to amplify upstream of start codon (included) and downstream of stop codon (included) of *adhE1* to conserve P1 promoter and ORF L and also to amplify entire 60bp between stop codon of *adhE1* and start codon of *ctfA.*

DNA fragments adhE1-1/adhE1-2 and adhE1-3/adhE1-4 were joined in a low cycle PCR fusion with Phusion DNA polymerase and primers adhE1-1 and adhE1-4, and the resulting fragment was cloned into the Zero Blunt TOPO vector to generate the TOPO-DadhE1 plasmid. The 1.2 kb *StuI* fragment from the previously described pSCB-FRT-catP*carrying the FRT-Pptb-catP* cassette was introduced at the unique *StuI* site of TOPO-DadhE1, to generate the TOPO-DadhE1-FRT-catP* plasmid.

The 3.5 kb *BglII* fragment from TOPO-DadhE1-FRT-catP* was then cloned into the *BamHI* digested pSOS95-upp (see above) to give the final pREP-Delta adhE1-catP*-upp plasmid.

The final constructed plasmid, pREP-Delta adhE1-catP*-upp, was introduced into *ΔCA_C1502 Δupp* strain to yield *ΔCA_C1502 Δupp ΔadhE1::catP* strain exerting a polar effect on *ctfAB*, parts of *sol* operon as well as *adhE1*, resulting in loss of acetone production ability.

In order to obtain the *catP* cassette (that contains transcriptional terminator) removed strain, pSOS95-upp-flp plasmid was introduced into *ΔCA_C1502 Δupp ΔadhE1::catP* strain. The continued polar effect on acetone formation in spite of the removal of *catP* cassette leaded to attempts to alter the location of *sol* operon promoter to downstream of the latter FRT site that is a putative transcriptional terminator. The plasmid, pREP-Delta adhE1-A1A4, for alteration of the location of *sol* promoter was constructed using the following procedure: a 1.3 kb FRT-Pptb-catP* fragment was amplified using the pREP-DadhE1-catP*-upp plasmid as template and the oligonucleotides AdhE1-A1 and AdhE1-A2 as primers, and a 6.5 kb fragment containing the *sol* promoter region was amplified using the *C. acetobutylicum* ATCC824 total gDNA as template and the oligonucleotides AdhE1-A3 and AdhE1-A4 as primers.

Both primers AdhE1-A2 and AdhE1-A3 have self-complementary 5’ extended sequences and the DNA fragments AdhE1-A-1/ AdhE1-A-2 and AdhE1-A-3/ AdhE1-A-4 were joined in a PCR fusion with primers AdhE1-A-1 and AdhE1-A-4.

Both primers AdhE1-A1 and AdhE1-A4 have 5’ extended sequence complementary to the pREP-Delta adhE1-catP-upp, thus after DpnI treatment, the resulting fused A1/A4 fragment was cloned into the pREP-Delta adhE1-catP-upp digested by *StuI* and *ClaI* using the GENEART Seamless Cloning and Assembly Kit (Invitrogen) to give the final pREP-Delta adhE1-A1A4 plasmid. This plasmid was then introduced into *ΔCA_C1502Δupp* strain to yield the *ΔCA_C1502ΔuppΔadhE1::catP-A1A4* strain.

***Deletion of adhE2***

For the *adhE2* (CA_P0035) replacement-deletion, the pREP-Delta adhE2-catP*-upp was constructed using the same procedures as for pREP-Delta adhE1-catP*-upp, excepted that the 1 kb upstream and 0.9 kb downstream homology regions immediately surrounding the *adhE2* gene (CA_P0035) were PCR amplified using pairs of primers adhE2-1/adhE2-2 and adhE2-3/adhE2-4, with *NruI* restriction site replacing *StuI* restriction site in the adhE2-2 and adhE2-3 primers.

**Table S1. Primers, strains, and plasmids used in this study**

| **Primer** | **Sequence** |
| --- | --- |
| Ldh-1 | aaaaggatccgctttaaaatttggaaagaggaagttgtg |
| Ldh-2 | ggggaggcctaaaaagggggttagaaatctttaaaaatttctctatagagcccatc |
| Ldh-3 | ccccctttttaggcctccccggtaaaagacctaaactccaagggtggaggctaggtc |
| Ldh-4 | aaaaggatcccccattgtggagaatattccaaagaagaaaataattgc |
| FRT-CM F1 | TACAGGCCTTGAGCGATTGTGTAGGCTGGAGCTGCTTCGAAGTTCCTATACTTTCTAGAGAATAGGAACTTCGGAATAGGAACTTCGGTTGGAATGGCGTGTGTGTTAGCCAAAGCTCCTGCAGGTCG |
| FRT-CM F2 | AACAGGCCTGGGATGTAACGCACTGAGAAGCCCATGGTCCATATGAATATCCTCCTTAGTTCCTATTCCGAAGTTCCTATTCTCTAGAAAGTATAGGAACTTCTCACACAGGAAACAGCTATGACCATG |
| REP-UPP F | AAAACAGCTGGGAGGAATGAAATAATGAGTAAAGTTACAC |
| REP-UPP R | AAAACAGCTGTTATTTTGTACCGAATAATCTATCTCCAGC |
| adhE1-0 | 5’-CCAGCCTAATGTAGGTATATCCTACG-3’ |
| adhE1-1 | AAAAAGATCTgctttagacgcagaacctgaaaaaccctc |
| adhE1-2 | GGGGAGGCCTAAAAAGGGGG**TTA**CATTTCTTGCGAGTAACAAGAGAATTTTTTTTGAGC |
| adhE1-3 | CCCCCTTTTTAGGCCTCCCCGCACTAGATGATCAATGCACAGGCGC |
| adhE1-4 | AAAAAGATCTGTAACATCTACGTGACCACCACGG |
| adhE1-5 | CATTTACTAAATCCATAGCTCCACCC |
| adhE1-A1 | TAAATTTAAAGATTTAGGCATAGAAATCGATGATAAAAAAATACTTAACGGAAAATTTTTAGTATAACTGGGATGTAACGCACTGAGAAGCCC |
| adhE1-A2 | CTTAATTTGTAGACTTCTGAAATAATACTACATTTGAGCGATTGTGTAGGCTGGAGCTGC |
| adhE1-A3 | ATGTAGTATTATTTCAGAAGTCTACAAATTAAG |
| adhE1-A4 | TAAAAAGTAGTTGAAATATGAAGGTTTACATAAATATACACTTCTTTCTAAAATATTTATTATATTTTAAAAATAATGTC |
| adhE1-3D | AACTATGGCAGGTATGGCATCCGC |
| adhE1-5R | GTCTTCAACTAAGCCCATACCGG |
| adhE2-0 | TATCTGGAAGCGGAAGTATAGGTGG |
| adhE2-1 | AAAAAGATCTAGATTTAATTGTAAGCGGCTCTTCCCG |
| adhE2-2 | GGGGTCGCGAAAAAAGGGGGTTATTCTTTTTGATTTGTAACTTTCATTTATATACACTCC |
| adhE2-3 | CCCCCTTTTTTCGCGACCCCGATAAAATGTCAGAGCTTGCTTTTGATGACC |
| adhE2-4 | AAAAAGATCTGGTGCTATTACAGGAACGCTTATGGC |
| adhE2-5 | GGGGTACATCAGCGTATATAAGACC |
| adhE2-3D | GAAGCATATGTTTCGGTTATGGCTACGG |
| adhE2-5R | TTCTTTCTTTAGCTGCGGCTATGGCAC |
| FLPI-D | AAAAGGATCCAAAAGGAGGGATTAAAATGCCACAATTTGGTATATTATGTAAAACACCACCT |
| FLPI-R | AAATGGCGCCGCGTACTTATATGCGTCTATTTATGTAGGATGAAAGGTA |

| **Strain or plasmid** | **Relevant characteristics** | **Source** |
| --- | --- | --- |
| ***C. acetobutylicum* ATCC 824** |  |  |
| *ΔCA_C1502Δupp* | Deletion of *upp* gene (CA_C2879) encoding uracil phosphoribosyl transferase and *CA_C1502* gene encoding the type II restriction endonuclease, control strain in this study |  |
| *ΔCA_C1502ΔuppΔadhE1::catP* | Replacement of *adhE1* gene (CA_P0162) by the *catP* cassette | This study |
| *ΔCA_C1502ΔuppΔadhE1* | Derived from *ΔCA_C1502 Δupp ΔadhE1::catP*, *catP* cassette removed | This study |
| *ΔCA_C1502ΔuppΔadhE1::catP-A1A4* | Derived from *ΔCA_C1502 ΔuppΔadhE1::catP*, sol operon promoter location changed from upstream of the latter FRT to downstream of that, used for the chemostats | This study |
| *ΔCA_C1502ΔuppΔadhE2::catP* | Replacement of *adhE2* gene (CA_P0035) by the *catP* cassette, used for the chemostats | This study |
| ***E. coli*** |  |  |
| Top10 |  | Invitrogen |
| **Plasmid** |  |  |
| pSOS95 |  |  |
| pSOS95-MLS^r^ | Acetone operon Pthl-ctfA-ctfB-adc eliminated, MLS^r^ |  |
| pSOS95-upp | Derived from pSOS95-MLS^r^, upp gene inserted |  |
| pREP-Delta adhE1-catP-upp | Derived from pSOS95-upp, adhE1-catP cassette inserted | This study |
| pSOS95-upp-flp S2 | Derived from pSOS95-upp, flp gene inserted |  |
| pREP-Delta adhE1-A1A4 | Derived from pREP-Delta adhE1-catP-upp, sol operon promoter location changed from upstream of the latter FRT to downstream of that | This study |
| pREP-Delta adhE2-catP-upp | Derived from pREPcel48A::upp-catP-11, adhE2-catP cassette inserted | This study |
| **Antibiotic resistance cassette** |  |  |
| FRT-CatP cassette | Amplified from pSOS94-Cm^c^ using primer FRT-CM F1 and FRT-CM F2 |  |

**Table S2. Four-fold increased or decreased genes under acidogenesis in *ΔadhE1***

| **Gene number** | **Function** | **adhE1**  **/Ctrl** | **Control** | **adhE1** |
| --- | --- | --- | --- | --- |
| **Increase** |  |  |  |  |
| **CAC0102** | O-acetylhomoserine sulfhydrylase | 28.7 | 0.06±0 | 1.79±0.75 |
| **CAC0103** | Adenylylsulfate kinase | 32.55 | 0.07±0 | 2.17±1.03 |
| **CAC0104** | Adenylylsulfate reductase, subunit A | 48.44 | 0.06±0 | 3.08±1.47 |
| **CAC0105** | Ferredoxin | 30.78 | 0.07±0 | 2.14±0.96 |
| **CAC0106** | ABC-type probable sulfate transporter, periplasmic binding protein | 26.09 | 0.12±0 | 3.07±1.56 |
| **CAC0107** | ABC-type sulfate transporter, ATPase component | 22.86 | 0.07±0.01 | 1.61±0.88 |
| **CAC0108** | ABC-type probable sulfate transporter, permease protein | 35.38 | 0.07±0 | 2.49±1.45 |
| **CAC0109** | Sulfate adenylate transferase, CysD subfamily | 42.53 | 0.08±0 | 3.59±2.17 |
| **CAC0110** | GTPase, sulfate adenylate transferase subunit 1 | 54.78 | 0.14±0.01 | 7.47±4.57 |
| **CAC0117** | Chemotaxis protein cheY homolog | 8.34 | 0.07±0 | 0.57±0.18 |
| **CAC0118** | Chemotaxis protein cheA | 11 | 0.07±0.01 | 0.78±0.25 |
| **CAC0119** | Chemotaxis protein cheW | 13.83 | 0.08±0.01 | 1.12±0.36 |
| **CAC0120** | Membrane-associated methyl-accepting chemotaxis protein with HAMP domain | 6.93 | 0.07±0 | 0.52±0.17 |
| **CAC0390** | Cystathionine gamma-synthase | 4.77 | 0.69±0.03 | 3.3±0.61 |
| **CAC0391** | Cystathionine beta-lyase | 4.6 | 0.26±0.01 | 1.19±0.15 |
| **CAC0422** | Transcriptional antiterminator licT | 4.72 | 1.08±0.27 | 5.09±2.35 |
| **CAC0423** | Fusion: PTS system, beta-glucosides specific IIABC component | 5.45 | 7.23±1.07 | 39.43±21.61 |
| **CAC0424** | Fructokinase | 5.59 | 2.8±0.18 | 15.65±7.99 |
| **CAC0425** | Sucrase-6-phosphate hydrolase (gene sacA) | 6.42 | 1.55±0.21 | 9.98±5.38 |
| **CAC0466** | Hypothetical protein | **∞** | 0±0 | 0.27±0.17 |
| **CAC0467** | Uncharacterized membrane protein, homolog of YDAH B.subtilis | 18.03 | 0.09±0 | 1.69±0.64 |
| **CAC0468** | HAD superfamily hydrolase | 20.33 | 0.1±0.01 | 1.94±0.93 |
| **CAC0751** | Permease | 9.95 | 0.57±0.03 | 5.67±0.06 |
| **CAC0818** | Diguanylate cyclase/phosphodiesterase domain (GGDEF) containing protein | 7.68 | 0.09±0.01 | 0.67±0.26 |
| **CAC0878** | Amino acid ABC transporter permease component | 5.61 | 0.13±0 | 0.7±0.34 |
| **CAC0879** | ABC-type polar amino acid transport system, ATPase component | 8.29 | 0.79±0.03 | 6.52±3.32 |
| **CAC0880** | Periplasmic amino acid binding protein | 9.5 | 0.68±0.06 | 6.44±3.18 |
| **CAC0930** | Cystathionine gamma-synthase | 4.58 | 0.13±0.04 | 0.61±0.14 |
| **CAC1031** | FeoB-like GTPase, responsible for iron uptake | 4.24 | 0.21±0.01 | 0.89±0.13 |
| **CAC1032** | Predicted transcriptional regulator | 4.44 | 0.13±0.01 | 0.59±0.2 |
| **CAC1353** | Phosphotransferase system IIC component, possibly N-acetylglucosamine-specific | 5.55 | 0.3±0.02 | 1.68±0.36 |
| **CAC1387** | Membrane associated chemotaxis sensory transducer protein (MSP domain and HAMP domain) | 10.86 | 0.17±0.01 | 1.84±0.64 |
| **CAC1392** | Glutamine phosphoribosylpyrophosphate amidotransferase | 4.2 | 0.53±0.03 | 2.21±0.23 |
| **CAC1394** | Folate-dependent phosphoribosylglycinamide formyltransferase | 4.11 | 0.34±0.02 | 1.39±0.08 |
| **CAC1405** | Beta-glucosidase | 5.16 | 6±0.61 | 30.95±15.6 |
| **CAC1406** | Transcriptional antiterminator (BglG family) | 4.41 | 11.33±2.2 | 49.91±22.5 |
| **CAC1407** | PTS system, beta-glucosides-specific IIABC component | 13.76 | 0.29±0.04 | 4.03±2.51 |
| **CAC1408** | Phospho-beta-glucosidase | 15.57 | 0.39±0.06 | 6.11±3.77 |
| **CAC1524** | Methyl-accepting chemotaxis-like domain (chemotaxis sensory transducer) | 8.14 | 0.07±0 | 0.6±0.21 |
| **CAC1525** | Uncharacterized protein, homolog of PHNB E.coli | 8.72 | 0.07±0 | 0.65±0.23 |
| **CAC1862** | Hypothetical protein | 6.54 | 0.14±0.01 | 0.89±0.31 |
| **CAC1863** | Hypothetical protein | 10.43 | 0.07±0 | 0.75±0.31 |
| **CAC2072** | Stage IV sporulation protein B, SpoIVB | ∞ | 0±0 | 0.39±0.03 |
| **CAC2235** | Cysteine synthase/cystathionine beta-synthase, CysK | 8.27 | 3.22±0.22 | 26.61±5.08 |
| **CAC2236** | Uncharacterized conserved protein of YjeB/RRF2 family | 4.29 | 2.22±0.49 | 9.5±0.84 |
| **CAC2241** | Cation transport P-type ATPase | 7.92 | 0.44±0.04 | 3.51±0.95 |
| **CAC2242** | Predicted transcriptional regulator, arsE family | 5.01 | 0.15±0.03 | 0.74±0.11 |
| **CAC2521** | Hypothetical protein, CF-41 family | 4.33 | 0.21±0.01 | 0.9±0.07 |
| **CAC2533** | Protein containing ChW-repeats | ∞ | 0±0 | 0.72±0.26 |
| **CAC2585** | 6-pyruvoyl-tetrahydropterin synthase related domain; conserved membrane protein | 17.69 | 0.07±0 | 1.25±0.4 |
| **CAC2586** | Predicted membrane protein | 19.16 | 0.07±0 | 1.25±0.44 |
| **CAC2587** | GGDEF domain containing protein | ∞ | 0±0 | 0.22±0.04 |
| **CAC2588** | Glycosyltransferase | 51.95 | 0.15±0.01 | 7.86±2.82 |
| **CAC2589** | Glycosyltransferase | 20.76 | 0.06±0 | 1.33±0.53 |
| **CAC2590** | Uncharacterized conserved membrane protein; | 28.44 | 0.06±0 | 1.77±0.69 |
| **CAC2591** | Hypothetical protein, CF-41 family | ∞ | 0±0 | 2.61±1.01 |
| **CAC2592** | 6-pyruvoyl-tetrahydropterin synthase related domain; conserved membrane protein | 28.02 | 0.09±0.01 | 2.39±1.02 |
| **CAC2605** | Transcriptional regulator (TetR/AcrR family) | 28.28 | 0.13±0.01 | 3.73±1.4 |
| **CAC2650** | Dihydroorotate dehydrogenase | 6.08 | 0.41±0.02 | 2.5±0.08 |
| **CAC2651** | Dihydroorotate dehydrogenase electron transfer subunit | 8.24 | 0.25±0.02 | 2.09±0.16 |
| **CAC2652** | Orotidine-5'-phosphate decarboxylase | 8.55 | 0.54±0.04 | 4.6±0.58 |
| **CAC2653** | Aspartate carbamoyltransferase regulatory subunit | 8.43 | 0.85±0.02 | 7.17±0.22 |
| **CAC2654** | Aspartate carbamoyltransferase catalytic subunit | 7.26 | 0.7±0.01 | 5.1±0.08 |
| **CAC2816** | Hypothetical protein, CF-17 family | 6 | 0.1±0 | 0.57±0.13 |
| **CAC2849** | Proline/glycine betaine ABC-type transport system, permease component fused to periplasmic component | 6.81 | 1.83±0.08 | 12.44±0.65 |
| **CAC2850** | Proline/glycine betaine ABC-type transport system, ATPase component | 6.96 | 1.74±0.19 | 12.12±0.56 |
| **CAC2937** | Ketopantoate reductase PanE/ApbA | 4.83 | 0.11±0 | 0.52±0.06 |
| **CAC3049** | Glycosyltransferase | 4.79 | 0.09±0 | 0.43±0.07 |
| **CAC3050** | AMSJ/WSAK related protein, possibly involved in exopolysaccharide biosynthesis | 4.7 | 0.11±0 | 0.5±0.09 |
| **CAC3051** | Glycosyltransferase | 5.16 | 0.11±0 | 0.55±0.09 |
| **CAC3052** | Glycosyltransferase | 5.59 | 0.12±0 | 0.65±0.11 |
| **CAC3053** | Histidinol phosphatase related enzyme | 7.03 | 0.17±0.01 | 1.16±0.18 |
| **CAC3054** | Phosphoheptose isomerase | 6.69 | 0.23±0.01 | 1.55±0.35 |
| **CAC3055** | Sugar kinase | 5.9 | 0.31±0.01 | 1.85±0.34 |
| **CAC3056** | Nucleoside-diphosphate-sugar pyrophosphorylase | 6.37 | 0.39±0.03 | 2.49±0.61 |
| **CAC3057** | Glycosyltransferase | 12.36 | 0.36±0.03 | 4.41±1.19 |
| **CAC3058** | Mannose-1-phosphate guanylyltransferase | 9.94 | 0.3±0.01 | 2.98±0.62 |
| **CAC3059** | Sugar transferases | 13.47 | 0.77±0.03 | 10.43±2.79 |
| **CAC3325** | Periplasmic amino acid binding protein | 18.24 | 0.11±0 | 1.93±0.82 |
| **CAC3326** | Amino acid ABC-type transporter, permease component | 19.82 | 0.11±0.01 | 2.11±0.98 |
| **CAC3327** | Amino acid ABC-type transporter, ATPase component | 28.33 | 0.56±0.1 | 15.77±7.65 |
| **CAC3461** | Hypothetical protein | 4.52 | 0.24±0.03 | 1.11±0.22 |
| **CAC3556** | Probable S-layer protein; | 4.18 | 1.92±0.24 | 8.04±1.07 |
| **CAC3636** | Oligopeptide ABC transporter, ATPase component | 4.23 | 0.97±0.07 | 4.11±1.17 |
| **CAC3647** | Transition state regulatory protein AbrB | 4.92 | 0.75±0.03 | 3.69±0.69 |
| **CAP0028** | HTH transcriptional regulator TetR family | 13.55 | 0.44±0.03 | 6.03±0.34 |
| **CAP0029** | Permease MDR-related | ∞ | 0±0 | 12.2±1.27 |
| **CAP0030** | Isochorismatase | 385.91 | 0.06±0 | 24.38±3.46 |
| **CAP0031** | Transcriptional activator HLYU, HTH of ArsR family | 46.17 | 0.69±0.38 | 32.04±4.76 |
| **CAP0032** | Rhodanese-like domain | 4.22 | 0.15±0.01 | 0.63±0.07 |
| **CAP0033** | Hypothetical protein | 4.76 | 0.91±0.03 | 4.35±0.48 |
| **CAP0035** | Aldehyde-alcohol dehydrogenase, ADHE1 | 5.44 | 0.42±0.02 | 2.31±0.6 |
| **CAP0071** | Possible xylan degradation enzyme (alpha/beta hydrolase domain and ricin-B-like domain) | 4.38 | 0.07±0 | 0.31±0.12 |
| **CAP0114** | Possible beta-xylosidase, family 43 of glycosyl hydrolases | 16.44 | 0.23±0.03 | 3.85±1.87 |
| **CAP0115** | Endo-1,4-beta-xylanase XynD B.subtilis ortholog (family 43 glycosyl hydrolase and cellulose-binding domain) | 19.51 | 0.3±0.03 | 5.9±2.78 |
| **CAP0116** | Xylanase, glycosyl hydrolase family 10 | 32.42 | 0.11±0.01 | 3.69±1.3 |
| **CAP0117** | Possible beta-xylosidase diverged, family 5/39 of glycosyl hydrolases and alpha-amylase C (Greek key) C-terminal domain | 56.53 | 0.24±0.03 | 13.65±4.85 |
| **CAP0118** | Possible xylan degradation enzyme (glycosyl hydrolase family 30-like domain and Ricin B-like domain) | 54.97 | 0.22±0.02 | 11.95±4.91 |
| **CAP0119** | Possible xylan degradation enzyme (glycosyl hydrolase family 30-like domain and Ricin B-like domain) | 46.44 | 0.12±0.01 | 5.59±2.18 |
| **CAP0120** | Possible xylan degradation enzyme (glycosyl hydrolase family 43-like domain, cellulose-binding domain and Ricin B-like domain) | 36.19 | 0.1±0.01 | 3.53±1.31 |
|  |  |  |  |  |
| **Decrease** |  |  |  |  |
| **CAC0029** | Distantly related to cell wall-associated hydrolases, similar to yycO Bacillus subtilis | 0.22 | 5.15±0.37 | 1.12±0.84 |
| **CAC0035** | Serine/threonine phosphatase (inactivated protein) | 0.25 | 1.57±0.06 | 0.39±0.18 |
| **CAC0078** | Accessory gene regulator protein B | 0.04 | 1.82±0.62 | 0.07±0.02 |
| **CAC0079** | Hypothetical protein | 0 | 40.95±4.74 | 0.19±0.19 |
| **CAC0082** | Predicted membrane protein | 0.02 | 40.84±3.37 | 0.8±0.66 |
| **CAC0141** | Membrane permease, predicted cation efflux pumps | 0.24 | 8.01±0.63 | 1.89±0.66 |
| **CAC0204** | Sortase (surface protein transpeptidase), YHCS B.subtilis ortholog | 0.18 | 3.65±0.24 | 0.66±0.29 |
| **CAC0205** | Predicted phosphohydrolases, Icc family | 0.21 | 16.4±0.6 | 3.48±3.13 |
| **CAC0206** | Uncharacterized conserved membrane protein | 0.17 | 5.06±0.47 | 0.84±0.42 |
| **CAC0310** | Regulators of stationary/sporulation gene expression, abrB B.subtilis ortholog | 0.15 | 7.79±3.79 | 1.14±0.52 |
| **CAC0353** | 2,3-cyclic-nucleotide 2'phosphodiesterase (duplication) | 0.19 | 2.19±0.05 | 0.43±0.29 |
| **CAC0381** | Methyl-accepting chemotaxis protein | 0.18 | 2.07±0.05 | 0.37±0.22 |
| **CAC0403** | Secreted protein contains fibronectin type III domains | 0.25 | 0.6±0.03 | 0.15±0.02 |
| **CAC0437** | Sensory transduction histidine kinase | 0.15 | 1.44±0.02 | 0.22±0.13 |
| **CAC0537** | Acetylxylan esterase, acyl-CoA esterase or GDSL lipase family, strong similarity to C-terminal region of endoglucanase E precursor | 0.15 | 20.85±1.01 | 3.07±1.79 |
| **CAC0542** | Methyl-accepting chemotaxis protein | 0.21 | 1.74±0.17 | 0.37±0.36 |
| **CAC0658** | Fe-S oxidoreductase | 0.24 | 0.73±0.04 | 0.18±0.03 |
| **CAC0660** | Hypothetical protein, CF-26 family | 0.17 | 5.73±0.37 | 0.95±0.24 |
| **CAC0746** | Secreted protease metal-dependent protease | 0.16 | 4.11±0.14 | 0.68±0.18 |
| **CAC0814** | 3-oxoacyl-[acyl-carrier-protein] synthase III | 0.11 | 6.25±0.26 | 0.72±0.43 |
| **CAC0815** | Methyl-accepting chemotaxis protein | 0.13 | 3.4±0.06 | 0.43±0.28 |
| **CAC0816** | Lipase-esterase related protein | 0.17 | 3.77±0.12 | 0.66±0.49 |
| **CAC0946** | ComE-like protein, Metallo beta-lactamase superfamily hydrolase, secreted | 0.18 | 7.6±0.56 | 1.35±1.22 |
| **CAC1010** | Predicted phosphohydrolase, Icc family | 0.21 | 6.5±0.44 | 1.37±0.85 |
| **CAC1022** | Thioesterase II of alpha/beta hydrolase superfamily | 0.22 | 0.87±0.03 | 0.19±0.12 |
| **CAC1078** | Predicted phosphohydrolase, Icc family | 0.17 | 6.77±0.47 | 1.18±0.74 |
| **CAC1079** | Uncharacterized protein, related to enterotoxins of other Clostridiales | 0.15 | 1.27±0.2 | 0.19±0.08 |
| **CAC1080** | Uncharacterized protein, probably surface-located | 0.11 | 20.76±0.39 | 2.37±1.73 |
| **CAC1081** | Uncharacterized protein, probably surface-located | 0.13 | 7.47±0.13 | 1.01±0.7 |
| **CAC1532** | Protein containing ChW-repeats | 0.22 | 1.98±0.08 | 0.44±0.25 |
| **CAC1766** | Predicted sigma factor | 0.19 | 0.34±0.03 | 0.06±0 |
| **CAC1775** | Predicted membrane protein | 0.16 | 5.53±0.37 | 0.87±0.61 |
| **CAC1868** | Uncharacterized secreted protein, homolog YXKC Bacillus subtilis | 0.22 | 1.01±0.1 | 0.22±0.14 |
| **CAC1989** | ABC-type iron (III) transport system, ATPase component | 0.18 | 2.78±0.1 | 0.5±0.18 |
| **CAC1991** | Uncharacterized protein, YIIM family | 0.23 | 1.66±0.1 | 0.39±0.15 |
| **CAC1993** | Molybdenum cofactor biosynthesis enzyme MoaA, Fe-S oxidoreductase | 0.23 | 0.45±0.02 | 0.1±0.04 |
| **CAC1994** | Molybdopterin biosynthesis enzyme, MoaB | 0.22 | 0.82±0.09 | 0.18±0.07 |
| **CAC1996** | Hypothetical protein | 0.19 | 1.45±0.16 | 0.28±0.12 |
| **CAC1997** | Predicted glycosyltransferase | 0.19 | 1.45±0.03 | 0.28±0.12 |
| **CAC1998** | ABC-type transport system, ATPase component | 0.19 | 1.31±0.1 | 0.24±0.13 |
| **CAC1999** | Uncharacterized protein related to hypothetical protein Cj1507c from Campylobacter jejuni | 0.2 | 1.14±0.07 | 0.23±0.12 |
| **CAC2000** | Indolepyruvate ferredoxin oxidoreductase, subunit beta | 0.19 | 1.48±0.05 | 0.27±0.15 |
| **CAC2001** | Indolepyruvate ferredoxin oxidoreductase, subunit alpha | 0.13 | 5.57±0.13 | 0.75±0.32 |
| **CAC2002** | Predicted iron-sulfur flavoprotein | 0.16 | 1.97±0.06 | 0.31±0.13 |
| **CAC2003** | Predicted permease | 0.16 | 0.89±0.02 | 0.14±0.05 |
| **CAC2004** | Siderophore/Surfactin synthetase related protein | 0.1 | 4.01±0.25 | 0.42±0.1 |
| **CAC2005** | Siderophore/Surfactin synthetase related protein | 0.12 | 2.22±0.3 | 0.27±0.08 |
| **CAC2006** | Enzyme of siderophore/surfactin biosynthesis | 0.15 | 0.96±0.19 | 0.15±0.05 |
| **CAC2007** | Predicted glycosyltransferase | 0.09 | 5.87±0.14 | 0.51±0.13 |
| **CAC2008** | 3-oxoacyl-(acyl-carrier-protein) synthase | 0.11 | 2.25±0.14 | 0.26±0.05 |
| **CAC2009** | 3-Hydroxyacyl-CoA dehydrogenase | 0.1 | 3.83±0.14 | 0.37±0.12 |
| **CAC2010** | Predicted Fe-S oxidoreductase | 0.09 | 5.38±0.16 | 0.49±0.2 |
| **CAC2011** | Possible 3-oxoacyl-[acyl-carrier-protein] synthase III | 0.12 | 3.32±0.16 | 0.41±0.15 |
| **CAC2012** | Enoyl-CoA hydratase | 0.12 | 2.31±0.07 | 0.28±0.13 |
| **CAC2013** | Hypothetical protein | 0.12 | 4.33±0.23 | 0.54±0.27 |
| **CAC2014** | Predicted esterase | 0.12 | 5.18±0.07 | 0.63±0.3 |
| **CAC2015** | Hypothetical protein | 0.15 | 2.28±0.08 | 0.33±0.16 |
| **CAC2016** | Enoyl-CoA hydratase | 0.12 | 13.81±0.63 | 1.7±0.83 |
| **CAC2017** | Acyl carrier protein | 0.15 | 3.51±0.12 | 0.51±0.29 |
| **CAC2018** | Aldehyde:ferredoxin oxidoreductase | 0.12 | 3.69±0.15 | 0.46±0.22 |
| **CAC2019** | Malonyl CoA-acyl carrier protein transacylase | 0.12 | 5.07±0.78 | 0.61±0.31 |
| **CAC2020** | Molybdopterin biosynthesis enzyme, MoeA, fused to molibdopterin-binding domain | 0.2 | 1.26±0.13 | 0.25±0.13 |
| **CAC2021** | Molybdopterin biosynthesis enzyme, MoeA (short form) | 0.24 | 2.88±0.54 | 0.7±0.28 |
| **CAC2023** | Membrane protein, related to copy number protein COP from Clostridium perfringens plasmid pIP404 (GI:116928) | 0.22 | 0.81±0.01 | 0.18±0.08 |
| **CAC2026** | Predicted flavodoxin | 0.2 | 3.83±0.2 | 0.77±0.49 |
| **CAC2107** | Contains cell adhesion domain | 0.2 | 0.87±0.03 | 0.18±0.15 |
| **CAC2293** | Hypothetical secreted protein | 0.13 | 2.47±0.26 | 0.31±0.23 |
| **CAC2517** | Extracellular neutral metalloprotease, NPRE | 0.17 | 1.63±0.16 | 0.27±0.07 |
| **CAC2518** | Extracellular neutral metalloprotease, NPRE (fragment or C-term. domain) | 0.22 | 1.53±0.37 | 0.33±0.18 |
| **CAC2581** | 6-pyruvoyl-tetrahydropterin synthase related domain; conserved membrane protein | 0.24 | 0.73±0.01 | 0.17±0.08 |
| **CAC2663** | Protein containing cell-wall hydrolase domain | 0.23 | 1.65±0.06 | 0.38±0.2 |
| **CAC2695** | Diverged Metallo-dependent hydrolase(Zn) of DD-Peptidase family; peptodoglycan-binding domain | 0.17 | 2.79±0.11 | 0.47±0.35 |
| **CAC2807** | Endo-1,3(4)-beta-glucanase family 16 | 0.21 | 78.48±1.92 | 16.84±17.3 |
| **CAC2808** | Beta-lactamase class C domain (PBPX family) containing protein | 0.2 | 2.67±0.25 | 0.53±0.27 |
| **CAC2809** | Predicted HD superfamily hydrolase | 0.14 | 4.61±0.4 | 0.66±0.33 |
| **CAC2810** | Possible glucoamylase (diverged), 15 family | 0.14 | 15.81±1.25 | 2.26±1.21 |
| **CAC2944** | N-terminal domain intergin-like repeats and c-terminal- cell wall-associated hydrolase domain | 0.23 | 5.72±0.45 | 1.32±0.61 |
| **CAC3070** | Glycosyltransferase | 0.21 | 4.34±0.23 | 0.9±0.81 |
| **CAC3071** | Glycosyltransferase | 0.21 | 5.54±0.28 | 1.15±1.04 |
| **CAC3072** | Mannose-1-phosphate guanylyltransferase | 0.18 | 9.16±0.51 | 1.6±1.49 |
| **CAC3073** | Sugar transferase involved in lipopolysaccharide synthesis | 0.23 | 4.21±0.85 | 0.96±0.91 |
| **CAC3085** | TPR-repeat-containing protein; Cell-adhesion domain; | 0.25 | 2.01±0.12 | 0.49±0.43 |
| **CAC3086** | Protein containing cell adhesion domain | 0.2 | 3.81±0.28 | 0.75±0.58 |
| **CAC3175** | Hypothetical protein | 0.21 | 3.62±2.52 | 0.76±0.12 |
| **CAC3251** | Sensory transduction protein containing HD_GYP domain | 0.2 | 1.91±0.03 | 0.39±0.27 |
| **CAC3264** | Uncharacterized conserved protein, YTFJ B.subtilis ortholog | 0.19 | 78.48±1.92 | 14.92±1.31 |
| **CAC3265** | Predicted membrane protein | 0.08 | 2.24±0.13 | 0.19±0.02 |
| **CAC3266** | Hypothetical protein | 0.07 | 8.71±0.16 | 0.63±0.03 |
| **CAC3267** | Specialized sigma subunit of RNA polymerase | 0.15 | 0.78±0.02 | 0.11±0 |
| **CAC3280** | Possible surface protein, responsible for cell interaction; contains cell adhesion domain and ChW-repeats | 0.23 | 0.55±0.07 | 0.13±0.05 |
| **CAC3408** | NADH oxidase (two distinct flavin oxidoreductase domains) | 0.03 | 5.91±0.22 | 0.16±0.07 |
| **CAC3409** | Transcriptional regulators, LysR family | 0.02 | 23.82±2.8 | 0.38±0.26 |
| **CAC3412** | Predicted protein-S-isoprenylcysteine methyltransferase | 0.22 | 1.55±0.04 | 0.33±0.19 |
| **CAC3422** | Sugar:proton symporter (possible xylulose) | 0.05 | 5.86±0.67 | 0.3±0.02 |
| **CAC3423** | Acetyltransferase (ribosomal protein N-acetylase subfamily) | 0.04 | 8.08±0.35 | 0.36±0.03 |
| **CAC3521** | Hypothetical protein | 0.14 | 8.82±0.24 | 1.23±0.46 |
| **CAC3522** | Hypothetical protein, CF-7 family | 0.14 | 6.64±0.43 | 0.95±0.29 |
| **CAC3523** | Hypothetical protein, CF-7 family | 0.15 | 2.36±0.17 | 0.36±0.08 |
| **CAC3524** | Hypothetical protein, CF-7 family | 0.19 | 2.35±0.08 | 0.45±0.11 |
| **CAC3558** | Probable S-layer protein; | 0.24 | 1.84±0.21 | 0.44±0.18 |
| **CAC3612** | Hypothetical protein | 0.18 | 0.85±0.07 | 0.16±0.05 |
| **CAP0053** | Xylanase, glycosyl hydrolase family 10 | 0.24 | 1.05±0.13 | 0.25±0.06 |
| **CAP0054** | Xylanase/chitin deacetylase family enzyme | 0.24 | 1.88±0.26 | 0.44±0.04 |
| **CAP0057** | Putative glycoportein or S-layer protein | 0.21 | 2.53±0.14 | 0.54±0.02 |
| **CAP0135** | Oxidoreductase | 0.25 | 16.08±0.99 | 3.94±2.61 |
| **CAP0136** | AstB/chuR/nirj-related protein | 0.25 | 2.99±0.1 | 0.74±0.42 |
| **CAP0148** | Phospholipase C | 0.22 | 1.04±0.06 | 0.23±0.11 |
| **CAP0174** | Membrane protein | 0.25 | 1.06±0.23 | 0.26±0.13 |

**Table S3**. Four-fold increased or decreased genes under acidogenesis in *ΔadhE2*

| **Gene number** | **Function** | **adhE2**  **/Ctrl** | **Control** | **adhE2** |
| --- | --- | --- | --- | --- |
| **Increase** |  |  |  |  |
| **CAC0040** | Uncharacterized small conserved protein, homolog of yfjA/yukE B.subtilis | 4.11 | 4.33±0.11 | 17.78±0.79 |
| **CAC0041** | Uncharacterized small conserved protein, homolog of yfjA/yukE B.subtilis | 4.14 | 0.1±0.01 | 0.42±0.06 |
| **CAC0042** | Hypothetical protein, CF-1 family | 5.71 | 0.93±0.02 | 5.34±0.19 |
| **CAC0043** | Hypothetical protein, CF-3 family | 5.79 | 0.54±0.03 | 3.12±0.29 |
| **CAC0044** | Predicted membrane protein | 5.49 | 0.86±0.06 | 4.71±0.28 |
| **CAC0045** | TPR-repeat-containing protein | 5.11 | 0.35±0.02 | 1.8±0.04 |
| **CAC0047** | Uncharacterized small conserved protein, homolog of yfjA/yukE B.subtilis | 4.91 | 0.77±0.03 | 3.79±0.12 |
| **CAC0048** | Hypothetical protein, CF-17 family | 5.19 | 0.73±0.03 | 3.79±0.17 |
| **CAC0049** | Hypothetical protein, CF-17 family | 4.18 | 0.14±0.02 | 0.59±0.11 |
| **CAC0056** | Hypothetical protein | 5.48 | 2.06±0.28 | 11.29±0.91 |
| **CAC0057** | Hypothetical protein | 5.29 | 5.97±0.54 | 31.56±1.37 |
| **CAC0058** | Hypothetical protein | 5.39 | 5.86±0.64 | 31.6±0.97 |
| **CAC0059** | Hypothetical protein | 5.48 | 2.89±0.14 | 15.81±1.9 |
| **CAC0060** | Predicted membrane protein | 4.96 | 1.93±0.07 | 9.58±0.56 |
| **CAC0061** | Phage-related protein, gp16 | 6.24 | 1.64±0.2 | 10.21±0.71 |
| **CAC0062** | Phage-related protein | 5.56 | 4.63±0.56 | 25.72±1.1 |
| **CAC0063** | Phage-related protein | 4.61 | 0.52±0.03 | 2.4±0.15 |
| **CAC0064** | Hypothetical protein | 4.39 | 0.96±0.08 | 4.23±0.29 |
| **CAC0065** | Hypothetical protein | 4.69 | 0.28±0.01 | 1.34±0.06 |
| **CAC0102** | O-acetylhomoserine sulfhydrylase | 20.49 | 0.06±0 | 1.28±0.08 |
| **CAC0103** | Adenylylsulfate kinase | 22.06 | 0.07±0 | 1.47±0.17 |
| **CAC0104** | Adenylylsulfate reductase, subunit A | 28.89 | 0.06±0 | 1.83±0.17 |
| **CAC0105** | Ferredoxin | 21.84 | 0.07±0 | 1.52±0.04 |
| **CAC0106** | ABC-type probable sulfate transporter, periplasmic binding protein | 14.54 | 0.12±0 | 1.71±0.05 |
| **CAC0107** | ABC-type sulfate transporter, ATPase component | 13.03 | 0.07±0.01 | 0.92±0.04 |
| **CAC0108** | ABC-type probable sulfate transporter, permease protein | 19.05 | 0.07±0 | 1.34±0.08 |
| **CAC0109** | Sulfate adenylate transferase, CysD subfamily | 26.82 | 0.08±0 | 2.26±0.04 |
| **CAC0110** | GTPase, sulfate adenylate transferase subunit 1 | 42.48 | 0.14±0.01 | 5.79±0.35 |
| **CAC0116** | Carbone-monoxide dehydrogenase, beta chain | 6.2 | 0.64±0.16 | 3.95±1.53 |
| **CAC0117** | Chemotaxis protein cheY homolog | 6.69 | 0.07±0 | 0.46±0.04 |
| **CAC0118** | Chemotaxis protein cheA | 8.24 | 0.07±0.01 | 0.58±0.06 |
| **CAC0119** | Chemotaxis protein cheW | 9.52 | 0.08±0.01 | 0.77±0.08 |
| **CAC0120** | Membrane-associated methyl-accepting chemotaxis protein with HAMP domain | 5.29 | 0.07±0 | 0.39±0.04 |
| **CAC0208** | Predicted membrane protein; CF-20 family | 11.53 | 0.51±0.03 | 5.84±0.18 |
| **CAC0209** | Predicted membrane protein; CF-20 family | 10.41 | 0.21±0.01 | 2.16±0.03 |
| **CAC0539** | Beta-mannanase ManB, contains ChW-repeats | 18.97 | 0.1±0 | 1.99±0.1 |
| **CAC0540** | Beta-mannanase ManB-like enzyme, contains ChW-repeats | 28.45 | 0.22±0 | 6.21±0.27 |
| **CAC0623** | Hypothetical protein | 5.18 | 0.28±0.03 | 1.48±0.19 |
| **CAC0682** | Ammonium transporter (membrane protein nrgA) | 8.97 | 0.24±0.01 | 2.17±0.16 |
| **CAC0706** | Endo-1,4-beta glucanase (fused to two ricin-B-like domains) | 7.5 | 1.19±0.13 | 8.92±0.84 |
| **CAC0754** | Hypothetical protein | 7.48 | 0.1±0.01 | 0.73±0.12 |
| **CAC0765** | Fe-S oxidoreductase | 25.78 | 0.14±0.01 | 3.54±0.11 |
| **CAC0766** | Predicted transcriptional regulator (MerR family) | 34.25 | 0.31±0.04 | 10.58±0.43 |
| **CAC0767** | Fe-S oxidoreductase | 15.02 | 0.59±0.05 | 8.87±0.25 |
| **CAC0771** | Cobalamin biosynthesis protein CbiM | 8.86 | 0.29±0.03 | 2.52±0.09 |
| **CAC0772** | Cobalt permease | 8.2 | 0.14±0.01 | 1.14±0.04 |
| **CAC0773** | ABC-type cobalt transport protein ATPase component | 7.31 | 0.12±0.01 | 0.88±0.04 |
| **CAC0774** | Uncharacterized conserved protein | 5.83 | 0.09±0 | 0.5±0.02 |
| **CAC0775** | ATP-utilizing enzyme of the PP-loop superfamily | 8.86 | 0.25±0.02 | 2.23±0.01 |
| **CAC0776** | NCAIR mutase (PurE)-related protein | 9.98 | 0.47±0.02 | 4.71±0.22 |
| **CAC0777** | Acetyltransferase (the isoleucine patch superfamily) | 8.01 | 0.17±0.01 | 1.34±0.07 |
| **CAC0878** | Amino acid ABC transporter permease component | 4.04 | 0.13±0 | 0.51±0.03 |
| **CAC0879** | ABC-type polar amino acid transport system, ATPase component | 5.6 | 0.79±0.03 | 4.4±0.45 |
| **CAC0880** | Periplasmic amino acid binding protein | 6.5 | 0.68±0.06 | 4.41±0.4 |
| **CAC0930** | Cystathionine gamma-synthase | 4.72 | 0.13±0.04 | 0.63±0.02 |
| **CAC0931** | Cysteine synthase | 4.26 | 0.08±0.01 | 0.34±0.02 |
| **CAC1357** | Uncharacterized predicted metal-binding protein | 5.86 | 1.11±0.07 | 6.49±1.14 |
| **CAC1392** | Glutamine phosphoribosylpyrophosphate amidotransferase | 4.47 | 0.53±0.03 | 2.34±0.25 |
| **CAC1393** | Phosphoribosylaminoimidazol (AIR) synthetase | 4.07 | 0.32±0.02 | 1.31±0.06 |
| **CAC1394** | Folate-dependent phosphoribosylglycinamide formyltransferase | 4.57 | 0.34±0.02 | 1.54±0.07 |
| **CAC2072** | Stage IV sporulation protein B, SpoIVB | ∞ | 0±0 | 0.4±0 |
| **CAC2235** | Cysteine synthase/cystathionine beta-synthase, CysK | 7.17 | 3.22±0.22 | 23.06±1.97 |
| **CAC2236** | Uncharacterized conserved protein of YjeB/RRF2 family | 4.06 | 2.22±0.49 | 8.99±0.85 |
| **CAC2241** | Cation transport P-type ATPase | 7.62 | 0.44±0.04 | 3.38±0.12 |
| **CAC2242** | Predicted transcriptional regulator, arsE family | 5.22 | 0.15±0.03 | 0.77±0.04 |
| **CAC2456** | Hypothetical protein, CF-40 family | 6.09 | 1.82±0.11 | 11.08±0.47 |
| **CAC2457** | Hypothetical protein | 6.48 | 2.06±0.18 | 13.34±1.5 |
| **CAC2521** | Hypothetical protein, CF-41 family | 5.7 | 0.21±0.01 | 1.18±0.04 |
| **CAC2533** | Protein containing ChW-repeats | ∞ | 0±0 | 0.31±0.03 |
| **CAC2534** | HD_GYP hydrolase domain fused to HD hydrolase domain | 5.26 | 0.1±0.02 | 0.52±0.04 |
| **CAC2548** | Reductase/isomerase/elongation factor common domain | 7.43 | 0.09±0 | 0.65±0.02 |
| **CAC2717** | Ethanolamine ammonia lyase small subunit | 4.54 | 0.1±0 | 0.43±0.02 |
| **CAC2718** | Ethanolamine ammonia lyase large subunit | 5.74 | 0.09±0.02 | 0.54±0 |
| **CAC2719** | Ethanolamin permease | ∞ | 0±0 | 0.26±0.01 |
| **CAC2720** | Sensory protein containing histidine kinase, PAS anf GAF domains | 4.43 | 0.24±0.01 | 1.07±0.06 |
| **CAC2816** | Hypothetical protein, CF-17 family | 11.2 | 0.1±0 | 1.07±0.02 |
| **CAC3013** | Hypothetical protein | 4.66 | 0.28±0.01 | 1.28±0.12 |
| **CAC3045** | CPSB/CAPC ortholog, PHP family hydrolase | 5.47 | 0.17±0.01 | 0.93±0.04 |
| **CAC3047** | Uncharacterized membrane protein, putative virulence factor MviN | 4.79 | 0.19±0 | 0.9±0.03 |
| **CAC3048** | Uncharacterized conserved membrane protein, possible transporter | 6.64 | 0.1±0.01 | 0.65±0.02 |
| **CAC3049** | Glycosyltransferase | 7.42 | 0.09±0 | 0.67±0.02 |
| **CAC3050** | AMSJ/WSAK related protein, possibly involved in exopolysaccharide biosynthesis | 8.25 | 0.11±0 | 0.88±0.02 |
| **CAC3051** | Glycosyltransferase | 9.6 | 0.11±0 | 1.01±0.13 |
| **CAC3052** | Glycosyltransferase | 9.91 | 0.12±0 | 1.16±0.05 |
| **CAC3053** | Histidinol phosphatase related enzyme | 10.94 | 0.17±0.01 | 1.81±0.13 |
| **CAC3054** | Phosphoheptose isomerase | 11.37 | 0.23±0.01 | 2.63±0.07 |
| **CAC3055** | Sugar kinase | 10.87 | 0.31±0.01 | 3.4±0.05 |
| **CAC3056** | Nucleoside-diphosphate-sugar pyrophosphorylase | 11.28 | 0.39±0.03 | 4.4±0.1 |
| **CAC3057** | Glycosyltransferase | 11.92 | 0.36±0.03 | 4.25±0.18 |
| **CAC3058** | Mannose-1-phosphate guanylyltransferase | 11.59 | 0.3±0.01 | 3.48±0.14 |
| **CAC3059** | Sugar transferases | 12.63 | 0.77±0.03 | 9.77±0.39 |
| **CAC3234** | Uncharacterized conserved protein, YVBJ B.subtilis ortholog with N-terminal C4-type Zn-finger domain | 15.12 | 0.26±0.03 | 3.95±0.07 |
| **CAC3235** | Uncharacterized conserved protein, YVBJ B.subtilis homolog | 10.9 | 0.12±0 | 1.36±0.01 |
| **CAC3236** | Possible transcriptional regulator from YAEG/LRPR family | 4.41 | 1.05±0.1 | 4.63±0.32 |
| **CAC3274** | Possible surface protein, responsible for cell interaction; contains cell adhesion domain and ChW-repeats | 16.99 | 0.32±0.04 | 5.44±0.22 |
| **CAC3275** | Possible surface protein, responsible for cell interaction; contains cell adhesion domain and ChW-repeats | 5.25 | 0.13±0.01 | 0.66±0.06 |
| **CAC3325** | Periplasmic amino acid binding protein | 10.68 | 0.11±0 | 1.13±0.05 |
| **CAC3326** | Amino acid ABC-type transporter, permease component | 11.79 | 0.11±0.01 | 1.25±0.07 |
| **CAC3327** | Amino acid ABC-type transporter, ATPase component | 16.73 | 0.56±0.1 | 9.31±0.53 |
| **CAC3357** | Hypothetical protein | 4.47 | 0.24±0.02 | 1.09±0.05 |
| **CAC3458** | Uncharacterized protein, homolog of B. anthracis (gi:48942631) | 17.16 | 0.49±0.03 | 8.37±0.16 |
| **CAC3459** | Homolog of cell division GTPase FtsZ, diverged | 26.29 | 0.6±0.05 | 15.88±1.22 |
| **CAC3461** | Hypothetical protein | 16.79 | 0.24±0.03 | 4.11±0.14 |
| **CAC3556** | Probable S-layer protein; | 10.41 | 1.92±0.24 | 19.99±0.98 |
| **CAC3583** | Predicted permease | 4.01 | 0.32±0.03 | 1.28±0.1 |
| **CAC3585** | ABC-type transporter, ATPase component | 4.94 | 1.29±0.06 | 6.35±0.29 |
| **CAC3604** | Dihydroxyacid dehydratase | 99.3 | 0.2±0.01 | 20.26±0.92 |
| **CAC3605** | High affinity gluconate/L-idonate permease | 83.89 | 0.13±0.01 | 11.11±2.07 |
| **CAC3635** | Oligopeptide ABC transporter, ATPase component | 4.02 | 0.69±0.03 | 2.76±0.12 |
| **CAC3636** | Oligopeptide ABC transporter, ATPase component | 4.68 | 0.97±0.07 | 4.55±0.3 |
| **CAC3650** | HD-GYP domain containing protein | 4.35 | 0.91±0.03 | 3.96±0.22 |
| **CAP0001** | Oxidoreductase | 5.9 | 0.11±0 | 0.64±0.01 |
| **CAP0029** | Permease MDR-related | ∞ | 0±0 | 2.44±0.1 |
| **CAP0030** | Isochorismatase | 81.89 | 0.06±0 | 5.17±0.11 |
| **CAP0031** | Transcriptional activator HLYU, HTH of ArsR family | 10.93 | 0.69±0.38 | 7.59±0.24 |
| **CAP0106** | 1-deoxyxylulose-5-phosphate synthase, dehydrogenase | 14.05 | 0.15±0 | 2.09±0.07 |
| **CAP0117** | Possible beta-xylosidase diverged, family 5/39 of glycosyl hydrolases and alpha-amylase C (Greek key) C-terminal domain | 4.94 | 0.24±0.03 | 1.19±0.06 |
| **CAP0118** | Possible xylan degradation enzyme (glycosyl hydrolase family 30-like domain and Ricin B-like domain) | 5.22 | 0.22±0.02 | 1.13±0.1 |
| **CAP0119** | Possible xylan degradation enzyme (glycosyl hydrolase family 30-like domain and Ricin B-like domain) | 4.23 | 0.12±0.01 | 0.51±0.05 |
|  |  |  |  |  |
| **Decrease** |  |  |  |  |
| **CAC0078** | Accessory gene regulator protein B | 0 | 1.82±0.62 | 0±0 |
| **CAC0079** | Hypothetical protein | 0 | 40.95±4.74 | 0.07±0 |
| **CAC0081** | Accessory gene regulator protein A | 0.13 | 0.72±0.03 | 0.09±0 |
| **CAC0082** | Predicted membrane protein | 0 | 40.84±3.37 | 0.19±0 |
| **CAC0086** | Muconate cycloisomerase related protein, ortholog of YKGB B.subtilis | 0.15 | 1.06±0.09 | 0.16±0.02 |
| **CAC0149** | Hypothetical protein | 0.12 | 5.36±0.15 | 0.65±0.05 |
| **CAC0154** | PTS system, mannitol-specific IIBC component (gene MtlA) | 0.21 | 1.39±0.31 | 0.29±0.07 |
| **CAC0155** | Putative regulator of the PTS system for mannitol (gene MltR) | 0.24 | 1.85±0.33 | 0.44±0.07 |
| **CAC0156** | PTS system, mannitol-specific IIA domain (Ntr-type) (gene MltF) | 0.22 | 6.45±0.37 | 1.44±0.07 |
| **CAC0193** | Uncharacterized conserved membrane protein, affecting LPS biosynthesis | 0.2 | 3.31±0.49 | 0.67±0.08 |
| **CAC0310** | Regulators of stationary/sporulation gene expression, abrB B.subtilis ortholog | 0.23 | 7.79±3.79 | 1.76±0.26 |
| **CAC0381** | Methyl-accepting chemotaxis protein | 0.13 | 2.07±0.05 | 0.27±0 |
| **CAC0437** | Sensory transduction histidine kinase | 0.23 | 1.44±0.02 | 0.33±0.03 |
| **CAC0537** | Acetylxylan esterase, acyl-CoA esterase or GDSL lipase family, strong similarity to C-terminal region of endoglucanase E precursor | 0.1 | 20.85±1.01 | 2.1±0.09 |
| **CAC0542** | Methyl-accepting chemotaxis protein | 0.08 | 1.74±0.17 | 0.14±0.01 |
| **CAC0543** | Methyl-accepting chemotaxis protein | 0.25 | 0.35±0.04 | 0.09±0 |
| **CAC0658** | Fe-S oxidoreductase | 0 | 0.73±0.04 | 0±0 |
| **CAC0659** | Predicted Zn-dependent peptidase | 0 | 0.52±0.09 | 0±0 |
| **CAC0660** | Hypothetical protein, CF-26 family | 0.08 | 5.73±0.37 | 0.48±0.11 |
| **CAC0663** | Hypothetical protein | 0.21 | 0.61±0.07 | 0.13±0.01 |
| **CAC0792** | D-amino acid aminotransferase | 0.15 | 1.47±0.14 | 0.23±0.01 |
| **CAC0804** | Pectate lyase related protein, secreted | 0 | 0.28±0.04 | 0±0 |
| **CAC0814** | 3-oxoacyl-[acyl-carrier-protein] synthase III | 0.02 | 6.25±0.26 | 0.13±0 |
| **CAC0815** | Methyl-accepting chemotaxis protein | 0.04 | 3.4±0.06 | 0.12±0 |
| **CAC0816** | Lipase-esterase related protein | 0.04 | 3.77±0.12 | 0.15±0 |
| **CAC1009** | Cell wall biogenesis enzyme (N-terminal domain related to N-Acetylmuramoyl-L-alanine amidase and C-terminal domain related to L-alanoyl-D-glutamate peptidase); peptodoglycan-binding domain | 0 | 0.24±0.03 | 0±0 |
| **CAC1010** | Predicted phosphohydrolase, Icc family | 0.04 | 6.5±0.44 | 0.26±0.01 |
| **CAC1022** | Thioesterase II of alpha/beta hydrolase superfamily | 0.11 | 0.87±0.03 | 0.09±0 |
| **CAC1072** | Fe-S oxidoreductase | 0 | 0.21±0.01 | 0±0 |
| **CAC1075** | Beta-glucosidase family protein | 0.1 | 0.93±0.13 | 0.09±0.01 |
| **CAC1078** | Predicted phosphohydrolase, Icc family | 0.04 | 6.77±0.47 | 0.26±0.02 |
| **CAC1079** | Uncharacterized protein, related to enterotoxins of other Clostridiales | 0.05 | 1.27±0.2 | 0.06±0 |
| **CAC1080** | Uncharacterized protein, probably surface-located | 0.01 | 20.76±0.39 | 0.12±0.01 |
| **CAC1081** | Uncharacterized protein, probably surface-located | 0.01 | 7.47±0.13 | 0.09±0.01 |
| **CAC1084** | Beta-glucosidase family protein | 0.13 | 1.02±0.29 | 0.13±0.04 |
| **CAC1085** | Alpha-glucosidase | 0.17 | 1.44±0.19 | 0.24±0.03 |
| **CAC1086** | Transcriptional regulators of NagC/XylR family | 0.15 | 2.76±0.2 | 0.41±0.01 |
| **CAC1102** | Predicted membrane protein | 0.16 | 8.87±1.24 | 1.43±0.2 |
| **CAC1365** | Cobalamin biosynthesis protein CbiM | 0.16 | 1.56±0.05 | 0.25±0.02 |
| **CAC1366** | Predicted membrane protein | 0.18 | 1.23±0.06 | 0.22±0.01 |
| **CAC1367** | Cobalt permease | 0.2 | 0.78±0.01 | 0.16±0.01 |
| **CAC1368** | Cobalt transport (ATPase component) | 0.18 | 1.23±0.11 | 0.22±0.01 |
| **CAC1369** | Histidinol-phosphate aminotransferase | 0.13 | 4.55±0.54 | 0.6±0.02 |
| **CAC1370** | Cobalamin biosynthesis protein CbiG | 0.16 | 1.84±0.04 | 0.3±0.01 |
| **CAC1371** | Possible kinase, diverged | 0.16 | 1.86±0.03 | 0.3±0.01 |
| **CAC1372** | Cobalamin biosynthesis enzyme CobT | 0.16 | 1.98±0.08 | 0.33±0.01 |
| **CAC1373** | Anaerobic Cobalt chelatase, cbiK | 0.17 | 1.35±0.05 | 0.24±0 |
| **CAC1374** | Cobyric acid synthase CbiP | 0.17 | 1.79±0.08 | 0.3±0.01 |
| **CAC1375** | Cobyrinic acid a,c-diamide synthase CobB | 0.2 | 0.78±0.04 | 0.16±0.02 |
| **CAC1376** | Precorrin isomerase, cbiC | 0.24 | 0.62±0.03 | 0.15±0 |
| **CAC1377** | Cobalamin biosynthesis protein CbiD | 0.17 | 2.52±0.11 | 0.43±0.01 |
| **CAC1381** | precorrin-6x reductase | 0.21 | 1.82±0.09 | 0.38±0.01 |
| **CAC1532** | Protein containing ChW-repeats | 0.08 | 1.98±0.08 | 0.15±0 |
| **CAC1580** | Hypothetical protein | 0.25 | 3.33±0.13 | 0.82±0.06 |
| **CAC1766** | Predicted sigma factor | 0 | 0.34±0.03 | 0±0 |
| **CAC1768** | Uncharacterized conserved protein, TraB family | 0.12 | 0.81±0.04 | 0.1±0 |
| **CAC1775** | Predicted membrane protein | 0.05 | 5.53±0.37 | 0.27±0.03 |
| **CAC1868** | Uncharacterized secreted protein, homolog YXKC Bacillus subtilis | 0.18 | 1.01±0.1 | 0.18±0 |
| **CAC1988** | Ferrichrome-binding periplasmic protein | 0.17 | 0.77±0.03 | 0.13±0 |
| **CAC1989** | ABC-type iron (III) transport system, ATPase component | 0.11 | 2.78±0.1 | 0.3±0.01 |
| **CAC1990** | ABC-type iron (III) transport system, permease component | 0.19 | 0.48±0.01 | 0.09±0 |
| **CAC1991** | Uncharacterized protein, YIIM family | 0.1 | 1.66±0.1 | 0.17±0.01 |
| **CAC1992** | Molybdenum cofactor biosynthesis enzyme, MoaC | 0.18 | 0.45±0.03 | 0.08±0 |
| **CAC1993** | Molybdenum cofactor biosynthesis enzyme MoaA, Fe-S oxidoreductase | 0.18 | 0.45±0.02 | 0.08±0.01 |
| **CAC1994** | Molybdopterin biosynthesis enzyme, MoaB | 0.11 | 0.82±0.09 | 0.09±0 |
| **CAC1995** | Hypothetical protein | 0 | 0.25±0.04 | 0±0 |
| **CAC1996** | Hypothetical protein | 0.08 | 1.45±0.16 | 0.11±0 |
| **CAC1997** | Predicted glycosyltransferase | 0.07 | 1.45±0.03 | 0.11±0 |
| **CAC1998** | ABC-type transport system, ATPase component | 0.07 | 1.31±0.1 | 0.1±0 |
| **CAC1999** | Uncharacterized protein related to hypothetical protein Cj1507c from Campylobacter jejuni | 0.07 | 1.14±0.07 | 0.08±0 |
| **CAC2000** | Indolepyruvate ferredoxin oxidoreductase, subunit beta | 0.06 | 1.48±0.05 | 0.1±0 |
| **CAC2001** | Indolepyruvate ferredoxin oxidoreductase, subunit alpha | 0.04 | 5.57±0.13 | 0.2±0 |
| **CAC2002** | Predicted iron-sulfur flavoprotein | 0.05 | 1.97±0.06 | 0.09±0 |
| **CAC2003** | Predicted permease | 0.08 | 0.89±0.02 | 0.07±0 |
| **CAC2004** | Siderophore/Surfactin synthetase related protein | 0.04 | 4.01±0.25 | 0.16±0 |
| **CAC2005** | Siderophore/Surfactin synthetase related protein | 0.05 | 2.22±0.3 | 0.11±0.01 |
| **CAC2006** | Enzyme of siderophore/surfactin biosynthesis | 0.07 | 0.96±0.19 | 0.07±0 |
| **CAC2007** | Predicted glycosyltransferase | 0.03 | 5.87±0.14 | 0.16±0.01 |
| **CAC2008** | 3-oxoacyl-(acyl-carrier-protein) synthase | 0.04 | 2.25±0.14 | 0.08±0 |
| **CAC2009** | 3-Hydroxyacyl-CoA dehydrogenase | 0.03 | 3.83±0.14 | 0.1±0.01 |
| **CAC2010** | Predicted Fe-S oxidoreductase | 0.03 | 5.38±0.16 | 0.14±0 |
| **CAC2011** | Possible 3-oxoacyl-[acyl-carrier-protein] synthase III | 0.03 | 3.32±0.16 | 0.11±0 |
| **CAC2012** | Enoyl-CoA hydratase | 0.04 | 2.31±0.07 | 0.09±0 |
| **CAC2013** | Hypothetical protein | 0.03 | 4.33±0.23 | 0.12±0.01 |
| **CAC2014** | Predicted esterase | 0.02 | 5.18±0.07 | 0.13±0 |
| **CAC2015** | Hypothetical protein | 0.04 | 2.28±0.08 | 0.08±0 |
| **CAC2016** | Enoyl-CoA hydratase | 0.02 | 13.81±0.63 | 0.26±0.02 |
| **CAC2017** | Acyl carrier protein | 0.03 | 3.51±0.12 | 0.09±0.01 |
| **CAC2018** | Aldehyde:ferredoxin oxidoreductase | 0.03 | 3.69±0.15 | 0.11±0.01 |
| **CAC2019** | Malonyl CoA-acyl carrier protein transacylase | 0.02 | 5.07±0.78 | 0.1±0.01 |
| **CAC2020** | Molybdopterin biosynthesis enzyme, MoeA, fused to molibdopterin-binding domain | 0.07 | 1.26±0.13 | 0.08±0.01 |
| **CAC2021** | Molybdopterin biosynthesis enzyme, MoeA (short form) | 0.06 | 2.88±0.54 | 0.16±0.03 |
| **CAC2022** | Molybdopterin biosynthesis enzyme, moaB | 0.08 | 1.84±0.18 | 0.15±0.01 |
| **CAC2023** | Membrane protein, related to copy number protein COP from Clostridium perfringens plasmid pIP404 (GI:116928) | 0.12 | 0.81±0.01 | 0.1±0 |
| **CAC2024** | Phosphatidylglycerophosphate synthase related protein (fragment) | 0.1 | 1.22±0.06 | 0.13±0.01 |
| **CAC2025** | Hypothetical protein | 0.09 | 3.61±0.51 | 0.31±0.02 |
| **CAC2026** | Predicted flavodoxin | 0.09 | 3.83±0.2 | 0.33±0.02 |
| **CAC2040** | ABC transported MDR-type, ATPase component | 0.23 | 0.48±0.04 | 0.11±0.01 |
| **CAC2107** | Contains cell adhesion domain | 0.08 | 0.87±0.03 | 0.07±0 |
| **CAC2226** | Enzyme of ILVE/PABC family (branched-chain amino acid aminotransferase/4-amino-4-deoxychorismate lyase) | 0.19 | 7.98±0.85 | 1.53±0.07 |
| **CAC2252** | Alpha-glucosidase fused to unknown alpha-amylase C-terminal. domain | 0.04 | 78.48±1.92 | 3.17±0.27 |
| **CAC2287** | Acyl-CoA reductase LuxC | 0.21 | 0.71±0.08 | 0.15±0.01 |
| **CAC2288** | Acyl-protein synthetase, luxE | 0.19 | 0.94±0.12 | 0.18±0 |
| **CAC2289** | Biotin carboxyl carrier protein | 0 | 0.39±0 | 0±0 |
| **CAC2293** | Hypothetical secreted protein | 0.1 | 2.47±0.26 | 0.26±0.03 |
| **CAC2382** | Single-strand DNA-binding protein, ssb | 0.15 | 0.68±0.03 | 0.1±0.01 |
| **CAC2514** | Beta galactosidase | 0.24 | 0.54±0.01 | 0.13±0 |
| **CAC2580** | Hypothetical protein, CF-41 family | 0 | 0.2±0.01 | 0±0 |
| **CAC2581** | 6-pyruvoyl-tetrahydropterin synthase related domain; conserved membrane protein | 0.11 | 0.73±0.01 | 0.08±0.01 |
| **CAC2584** | Protein containing ChW-repeats | 0.16 | 0.47±0.01 | 0.08±0 |
| **CAC2597** | Hypothetical protein | 0.24 | 1.04±0.02 | 0.25±0 |
| **CAC2610** | L-fucose isomerase related protein | 0.23 | 0.74±0.11 | 0.17±0.01 |
| **CAC2611** | Hypothetical protein | 0.24 | 0.74±0.06 | 0.18±0.02 |
| **CAC2663** | Protein containing cell-wall hydrolase domain | 0.09 | 1.65±0.06 | 0.15±0.01 |
| **CAC2695** | Diverged Metallo-dependent hydrolase(Zn) of DD-Peptidase family; peptodoglycan-binding domain | 0.12 | 2.79±0.11 | 0.33±0.04 |
| **CAC2722** | RCC1 repeats protein (beta propeller fold) | 0.19 | 1.01±0.02 | 0.19±0.02 |
| **CAC2805** | Possible selenocysteine lyase (aminotransferase of NifS family) | 0.1 | 0.83±0.07 | 0.08±0 |
| **CAC2806** | Predicted phosphohydrolase, Icc family | 0.08 | 78.48±1.92 | 6.56±0.11 |
| **CAC2807** | Endo-1,3(4)-beta-glucanase family 16 | 0.02 | 78.48±1.92 | 1.62±0.18 |
| **CAC2808** | Beta-lactamase class C domain (PBPX family) containing protein | 0.04 | 2.67±0.25 | 0.09±0 |
| **CAC2809** | Predicted HD superfamily hydrolase | 0.02 | 4.61±0.4 | 0.08±0 |
| **CAC2810** | Possible glucoamylase (diverged), 15 family | 0.01 | 15.81±1.25 | 0.2±0.03 |
| **CAC2943** | N-terminal domain intergin-like repeats and c-terminal - cell wall-associated hydrolase domain | 0.14 | 0.53±0.05 | 0.07±0 |
| **CAC2944** | N-terminal domain intergin-like repeats and c-terminal- cell wall-associated hydrolase domain | 0.06 | 5.72±0.45 | 0.35±0.02 |
| **CAC3060** | CPSC/CAPB subfamily ATPase | 0.24 | 1.6±0.06 | 0.39±0.02 |
| **CAC3066** | Glycosyltransferase | 0.13 | 0.95±0.06 | 0.13±0.01 |
| **CAC3067** | Predicted membrane protein | 0 | 0.29±0.03 | 0±0 |
| **CAC3068** | Glycosyltransferase | 0.1 | 0.8±0.05 | 0.08±0.01 |
| **CAC3069** | Predicted glycosyltransferase | 0.08 | 0.81±0.04 | 0.07±0 |
| **CAC3070** | Glycosyltransferase | 0.04 | 4.34±0.23 | 0.15±0 |
| **CAC3071** | Glycosyltransferase | 0.03 | 5.54±0.28 | 0.18±0.01 |
| **CAC3072** | Mannose-1-phosphate guanylyltransferase | 0.02 | 9.16±0.51 | 0.22±0 |
| **CAC3073** | Sugar transferase involved in lipopolysaccharide synthesis | 0.03 | 4.21±0.85 | 0.13±0 |
| **CAC3085** | TPR-repeat-containing protein; Cell-adhesion domain; | 0.12 | 2.01±0.12 | 0.24±0.02 |
| **CAC3086** | Protein containing cell adhesion domain | 0.11 | 3.81±0.28 | 0.43±0.03 |
| **CAC3251** | Sensory transduction protein containing HD_GYP domain | 0.11 | 1.91±0.03 | 0.2±0 |
| **CAC3264** | Uncharacterized conserved protein, YTFJ B.subtilis ortholog | 0.15 | 78.48±1.92 | 11.7±0.94 |
| **CAC3265** | Predicted membrane protein | 0.11 | 2.24±0.13 | 0.24±0.03 |
| **CAC3266** | Hypothetical protein | 0.07 | 8.71±0.16 | 0.65±0.02 |
| **CAC3267** | Specialized sigma subunit of RNA polymerase | 0.16 | 0.78±0.02 | 0.12±0 |
| **CAC3279** | Possible surface protein, responsible for cell interaction; contains cell adhesion domain and ChW-repeats | 0.19 | 0.36±0.03 | 0.07±0.01 |
| **CAC3280** | Possible surface protein, responsible for cell interaction; contains cell adhesion domain and ChW-repeats | 0.14 | 0.55±0.07 | 0.08±0.01 |
| **CAC3298** | NADH-dependent butanol dehydrogenase B (BDH II) | 0.09 | 16.31±0.45 | 1.52±0.11 |
| **CAC3319** | Signal transduction histidine kinase | 0.06 | 3.14±0.66 | 0.19±0.02 |
| **CAC3320** | Predicted secreted protein homolog of yjcM/yhbB B.subtilis | 0.08 | 1.41±0.1 | 0.11±0.01 |
| **CAC3355** | Polyketide synthase pksE (short-chain alcohol dehydrogenase,acyl-carrier-protein S-malonyltransferase,3-oxoacyl-(acyl-carrier-protein) synthase I domains) | 0 | 0.4±0.02 | 0±0 |
| **CAC3408** | NADH oxidase (two distinct flavin oxidoreductase domains) | 0.02 | 5.91±0.22 | 0.1±0 |
| **CAC3409** | Transcriptional regulators, LysR family | 0.01 | 23.82±2.8 | 0.13±0 |
| **CAC3411** | Homolog of plant auxin-responsive GH3-like protein | 0 | 0.39±0.01 | 0±0 |
| **CAC3412** | Predicted protein-S-isoprenylcysteine methyltransferase | 0.06 | 1.55±0.04 | 0.09±0 |
| **CAC3422** | Sugar:proton symporter (possible xylulose) | 0.03 | 5.86±0.67 | 0.17±0.03 |
| **CAC3423** | Acetyltransferase (ribosomal protein N-acetylase subfamily) | 0.03 | 8.08±0.35 | 0.22±0.02 |
| **CAC3565** | Uncharacterized secreted protein, containing cell adhesion domain | 0.14 | 0.7±0.05 | 0.1±0 |
| **CAC3566** | Hypothetical protein, CF-28 family | 0.13 | 0.81±0.1 | 0.1±0 |
| **CAC3612** | Hypothetical protein | 0 | 0.85±0.07 | 0±0 |
| **CAC3613** | Hypothetical protein | 0.21 | 0.32±0.04 | 0.07±0 |
| **CAP0028** | HTH transcriptional regulator TetR family | 0.19 | 0.44±0.03 | 0.08±0 |
| **CAP0035** | Aldehyde-alcohol dehydrogenase, ADHE1 | 0 | 0.42±0.02 | 0±0 |
| **CAP0053** | Xylanase, glycosyl hydrolase family 10 | 0.09 | 1.05±0.13 | 0.1±0.01 |
| **CAP0054** | Xylanase/chitin deacetylase family enzyme | 0.07 | 1.88±0.26 | 0.14±0.01 |
| **CAP0057** | Putative glycoportein or S-layer protein | 0.13 | 2.53±0.14 | 0.33±0 |
| **CAP0058** | Rare lipoprotein A RLPA releated protein | 0.05 | 6.1±0.36 | 0.3±0.03 |
| **CAP0072** | Hypothetical protein | 0.09 | 1.45±0.08 | 0.13±0 |
| **CAP0098** | Alpha-amylase, AmyB | 0.19 | 1.38±0.17 | 0.26±0.02 |
| **CAP0135** | Oxidoreductase | 0.21 | 16.08±0.99 | 3.34±0.18 |
| **CAP0136** | AstB/chuR/nirj-related protein | 0.23 | 2.99±0.1 | 0.69±0.03 |
| **CAP0137** | Similar to C-ter. fragment of UDP-glucuronosyltransferases, YpfP B.subtilis related | 0.21 | 5.84±0.33 | 1.23±0.05 |
| **CAP0138** | Diverged, distantly related to biotin carboxylase N-term. fragment. | 0.25 | 5.38±0.07 | 1.33±0.07 |
| **CAP0160** | Secreted protein containing cell-adhesion domains | 0.2 | 0.54±0.07 | 0.11±0.01 |
| **CAP0174** | Membrane protein | 0.14 | 1.06±0.23 | 0.15±0 |

**Table S4. Four-fold increased or decreased genes under solventogenesis in *ΔadhE1***

| **Gene number** | **Function** | **adhE1**  **/Ctrl** | **Control** | **adhE1** |
| --- | --- | --- | --- | --- |
| **Increase** |  |  |  |  |
| **CAC0102** | O-acetylhomoserine sulfhydrylase | 32.98 | 0.13±0.03 | 4.2±0.26 |
| **CAC0103** | Adenylylsulfate kinase | 50.51 | 0.1±0.02 | 5.14±0.31 |
| **CAC0104** | Adenylylsulfate reductase, subunit A | 64.43 | 0.12±0.02 | 7.62±0.27 |
| **CAC0105** | Ferredoxin | 44.64 | 0.14±0.03 | 6.36±0.07 |
| **CAC0106** | ABC-type probable sulfate transporter, periplasmic binding protein | 18.89 | 0.5±0.17 | 9.4±0.36 |
| **CAC0107** | ABC-type sulfate transporter, ATPase component | 41.92 | 0.11±0.01 | 4.52±0.13 |
| **CAC0108** | ABC-type probable sulfate transporter, permease protein | 52.22 | 0.13±0.02 | 6.53±0.46 |
| **CAC0109** | Sulfate adenylate transferase, CysD subfamily | 44.6 | 0.2±0.05 | 8.76±0.49 |
| **CAC0110** | GTPase, sulfate adenylate transferase subunit 1 | 30.99 | 0.68±0.31 | 21.08±0.96 |
| **CAC0241** | ABC-type multidrug transport system, ATP-ase compoment | 6.71 | 0.09±0.02 | 0.61±0.02 |
| **CAC0243** | Predicted permease | 6.87 | 0.11±0.01 | 0.79±0.03 |
| **CAC0267** | L-lactate dehydrogenase | 5.1 | 0.55±0.17 | 2.83±0.05 |
| **CAC0403** | Secreted protein contains fibronectin type III domains | 5.32 | 0.17±0.09 | 0.93±0.05 |
| **CAC0409** | Hypothetical protein | 4.71 | 0.49±0.27 | 2.32±0.09 |
| **CAC0718** | Ortholog ycnD B.subtilis, nitroreductase | 5.2 | 0.67±0.36 | 3.48±0.16 |
| **CAC0867** | Putative permease, ortholog yfkN B.subtilis | 4.69 | 0.52±0.11 | 2.46±0.05 |
| **CAC0879** | ABC-type polar amino acid transport system, ATPase component | 5 | 0.82±0.09 | 4.12±0.08 |
| **CAC0880** | Periplasmic amino acid binding protein | 4.67 | 0.86±0.05 | 4±0.17 |
| **CAC0930** | Cystathionine gamma-synthase | 4.1 | 0.12±0.01 | 0.5±0.05 |
| **CAC0931** | Cysteine synthase | 4.4 | 0.1±0.01 | 0.43±0.02 |
| **CAC1283** | Molecular chaperones DnaJ (HSP40 family) | 4.33 | 7.34±3.4 | 31.8±0.28 |
| **CAC1284** | SAM-dependent methyltransferase | 4.43 | 0.61±0.2 | 2.72±0.18 |
| **CAC1356** | Thiamine biosynthesis enzyme ThiH | 7.7 | 1.96±1.44 | 15.11±0.26 |
| **CAC1547** | Thioredoxin, trx | 7.8 | 0.23±0.01 | 1.81±0.05 |
| **CAC1548** | Thioredoxin reductase | 9.47 | 1±0.07 | 9.44±0.07 |
| **CAC1549** | Glutathione peroxidase | 9.14 | 0.69±0.07 | 6.28±0.42 |
| **CAC1570** | Glutathione peroxidase | 7.45 | 0.26±0.11 | 1.95±0.01 |
| **CAC1571** | Glutathione peroxidase | 6.24 | 0.23±0.11 | 1.45±0.11 |
| **CAC1656** | Hypothetical protein, CF-39 family | 6.53 | 0.61±0.42 | 3.97±0.06 |
| **CAC1695** | DNA-dependent RNA polymerase sigma subunit | 4.86 | 0.16±0.01 | 0.75±0.04 |
| **CAC1696** | Specialized DNA-dependent RNA polymerase sigma subunit | 4.67 | 0.12±0.01 | 0.55±0.03 |
| **CAC1766** | Predicted sigma factor | 4.44 | 0.12±0.01 | 0.52±0.05 |
| **CAC2235** | Cysteine synthase/cystathionine beta-synthase, CysK | 4.06 | 2.46±0.13 | 9.99±0.22 |
| **CAC2456** | Hypothetical protein, CF-40 family | 5 | 0.31±0.1 | 1.55±0.1 |
| **CAC2457** | Hypothetical protein | 4.64 | 0.35±0.11 | 1.61±0.09 |
| **CAC2536** | Glycosyltransferase | 4.17 | 0.26±0.07 | 1.07±0.08 |
| **CAC2605** | Transcriptional regulator (TetR/AcrR family) | 4.8 | 0.14±0.04 | 0.66±0.02 |
| **CAC2906** | Spore coat protein cotS related | 4.9 | 0.06±0.02 | 0.32±0.02 |
| **CAC2991** | Methionyl-tRNA synthetase | 4.23 | 0.86±0.23 | 3.65±0.18 |
| **CAC3258** | Hypothetical protein | 4.58 | 0.16±0.05 | 0.71±0 |
| **CAC3266** | Hypothetical protein | 4.37 | 0.86±0.22 | 3.74±0.11 |
| **CAC3325** | Periplasmic amino acid binding protein | 10.14 | 0.32±0.11 | 3.21±0.05 |
| **CAC3326** | Amino acid ABC-type transporter, permease component | 11.64 | 0.32±0.11 | 3.73±0.15 |
| **CAC3327** | Amino acid ABC-type transporter, ATPase component | 10.54 | 2.56±1.07 | 26.98±2.27 |
| **CAC3550** | Na+ ABC transporter, NATB | 8.72 | 0.2±0.03 | 1.77±0.1 |
| **CAC3551** | Na+ ABC transporter (ATP-binding protein), NATA | 5.22 | 0.12±0.01 | 0.6±0.06 |
| **CAC3677** | KDP operon transcriptional regulatory protein KdpE (CheY-like receiver domain and HTH-type DNA-binding domain) | 7.61 | 1.06±0.06 | 8.1±0.38 |
| **CAC3678** | Sensor protein KdpD (ATPase containing sensor domain and histidine kinase domain) | 20.12 | 0.38±0.07 | 7.71±0.14 |
| **CAC3679** | Uncharacterized protein of kdp operon, kdpX | 32.53 | 0.61±0.26 | 20±0.67 |
| **CAC3680** | K+-transporting ATPase, c chain | 34.53 | 0.54±0.27 | 18.73±0.73 |
| **CAC3681** | K+-transporting ATPase, b chain | 32.53 | 0.2±0.09 | 6.65±0.36 |
| **CAC3682** | K+-transporting ATPase, a chain | 36.85 | 0.39±0.19 | 14.28±1.24 |
| **CAP0035** | Aldehyde-alcohol dehydrogenase, ADHE1 | 125.83 | 0.21±0.02 | 26.6±0.26 |
| **CAP0044** | Hypothetical protein | 7.95 | 0.37±0.11 | 2.93±0.15 |
| **CAP0045** | Glycosyl transferase | 10.81 | 1.03±0.4 | 11.16±0.51 |
|  |  |  |  |  |
| **Decrease** |  |  |  |  |
| **CAC0086** | Muconate cycloisomerase related protein, ortholog of YKGB B.subtilis | 0.17 | 2.27±0.3 | 0.38±0.01 |
| **CAC0149** | Hypothetical protein | 0.02 | 2.83±1.44 | 0.06±0 |
| **CAC0154** | PTS system, mannitol-specific IIBC component (gene MtlA) | 0.09 | 0.93±0.44 | 0.08±0 |
| **CAC0155** | Putative regulator of the PTS system for mannitol (gene MltR) | 0.07 | 1.32±0.61 | 0.1±0 |
| **CAC0156** | PTS system, mannitol-specific IIA domain (Ntr-type) (gene MltF) | 0.08 | 3.3±1.76 | 0.27±0.02 |
| **CAC0157** | Mannitol-1-phosphate 5-dehydrogenase (gene MtlD) | 0.1 | 1.26±0.72 | 0.12±0.01 |
| **CAC0164** | ABC transporter, ATP binding-protein | 0.07 | 2.24±0.92 | 0.15±0.01 |
| **CAC0165** | Predicted ABC transporter, permease component | 0.09 | 2.03±0.76 | 0.18±0.02 |
| **CAC0392** | Peptodoglycan-binding domain | 0.23 | 0.65±0.11 | 0.15±0.01 |
| **CAC0427** | Glycerol-3-phosphate ABC-transporter, permease component | 0.18 | 2.11±0.42 | 0.39±0.02 |
| **CAC0428** | Sugar permease | 0.21 | 16.27±3.86 | 3.38±0.1 |
| **CAC0435** | Hypothetical protein | 0.21 | 0.36±0.2 | 0.07±0.01 |
| **CAC0542** | Methyl-accepting chemotaxis protein | 0.21 | 3.47±0.15 | 0.73±0.05 |
| **CAC0553** | Hypothetical protein, CF-8 family | 0.22 | 4.72±1.57 | 1.03±0.02 |
| **CAC0554** | Autolytic lysozime (1,4-beta-N-acetylmuramidase), family 25 of glycosyl hydrolases ; peptodoglycan-binding domain | 0.21 | 2.37±0.71 | 0.51±0.01 |
| **CAC0706** | Endo-1,4-beta glucanase (fused to two ricin-B-like domains) | 0.22 | 0.49±0.2 | 0.11±0.01 |
| **CAC0707** | RNA polymerase sigma-54 factor | 0.21 | 2.88±0.5 | 0.6±0.02 |
| **CAC0751** | Permease | 0.14 | 0.95±0.61 | 0.13±0.01 |
| **CAC0814** | 3-oxoacyl-[acyl-carrier-protein] synthase III | 0.23 | 7.59±1.03 | 1.74±0.12 |
| **CAC0815** | Methyl-accepting chemotaxis protein | 0.22 | 4.32±0.19 | 0.95±0.01 |
| **CAC0816** | Lipase-esterase related protein | 0.2 | 5.09±0.55 | 0.99±0.11 |
| **CAC1075** | Beta-glucosidase family protein | 0.05 | 2.2±0.63 | 0.11±0.01 |
| **CAC1078** | Predicted phosphohydrolase, Icc family | 0.23 | 6.91±3.39 | 1.59±0.05 |
| **CAC1079** | Uncharacterized protein, related to enterotoxins of other Clostridiales | 0.04 | 2.62±1.06 | 0.11±0.01 |
| **CAC1080** | Uncharacterized protein, probably surface-located | 0.03 | 18.01±8.43 | 0.55±0.01 |
| **CAC1081** | Uncharacterized protein, probably surface-located | 0.03 | 8.4±4.15 | 0.25±0.02 |
| **CAC1084** | Beta-glucosidase family protein | 0.08 | 1.21±0.63 | 0.09±0.01 |
| **CAC1085** | Alpha-glucosidase | 0.08 | 1.33±0.72 | 0.11±0.01 |
| **CAC1086** | Transcriptional regulators of NagC/XylR family | 0.09 | 2.31±1.16 | 0.2±0.01 |
| **CAC1231** | Predicted dehydrogenase, YULF B.subtilis ortholog | 0.09 | 2.51±0.87 | 0.23±0.02 |
| **CAC1232** | Predicted lytic murein transglycosylase (N-term. LysM motif repeat domain) | 0.08 | 1.47±0.57 | 0.12±0.02 |
| **CAC1319** | Glycerol uptake facilitator protein, GLPF | 0 | 0.4±0.09 | 0±0 |
| **CAC1320** | Glycerol-3-phosphate responsive antiterminator (mRNA-binding), GLPP | 0 | 0.25±0.03 | 0±0 |
| **CAC1321** | Glycerol kinase, GLPK | 0 | 0.39±0.11 | 0±0 |
| **CAC1322** | Glycerol-3-phosphate dehydrogenase, GLPA | 0.16 | 0.57±0.03 | 0.09±0 |
| **CAC1323** | NAD(FAD)-dependent dehydrogenase | 0.19 | 0.4±0.03 | 0.08±0 |
| **CAC1324** | Uncharacterized predected metal-binding protein | 0.05 | 1.23±1.23 | 0.07±0 |
| **CAC1346** | L-arabinose isomerase | 0 | 0.21±0.07 | 0±0 |
| **CAC1349** | Aldose-1-epimerase | 0.25 | 1.76±1.22 | 0.43±0.01 |
| **CAC1405** | Beta-glucosidase | 0.15 | 36.33±12.49 | 5.38±0.16 |
| **CAC1406** | Transcriptional antiterminator (BglG family) | 0.04 | 3.1±1.96 | 0.14±0.01 |
| **CAC1436** | Hypothetical protein | 0.18 | 1.49±0.62 | 0.27±0.01 |
| **CAC1454** | Membrane associated histidine kinase-like ATPase | 0.22 | 0.57±0.32 | 0.13±0 |
| **CAC1455** | Two-component system regulator (CheY domain and HTH-like DNA-binding domain) | 0.21 | 1.68±0.88 | 0.36±0 |
| **CAC1669** | Carbon starvation protein | 0.1 | 2.67±0.5 | 0.27±0.01 |
| **CAC1775** | Predicted membrane protein | 0.09 | 8.38±1.21 | 0.72±0.05 |
| **CAC1909** | Ribonuclease D | 0.22 | 0.31±0.13 | 0.07±0.01 |
| **CAC1988** | Ferrichrome-binding periplasmic protein | 0.21 | 1.98±0.61 | 0.41±0.03 |
| **CAC1989** | ABC-type iron (III) transport system, ATPase component | 0.23 | 5.22±1.52 | 1.22±0.08 |
| **CAC1990** | ABC-type iron (III) transport system, permease component | 0.23 | 0.98±0.26 | 0.23±0.01 |
| **CAC1991** | Uncharacterized protein, YIIM family | 0.23 | 3.03±1.07 | 0.7±0.02 |
| **CAC1993** | Molybdenum cofactor biosynthesis enzyme MoaA, Fe-S oxidoreductase | 0.2 | 0.96±0.37 | 0.2±0.01 |
| **CAC1994** | Molybdopterin biosynthesis enzyme, MoaB | 0.18 | 1.42±0.53 | 0.25±0.01 |
| **CAC1995** | Hypothetical protein | 0.22 | 0.46±0.19 | 0.1±0.02 |
| **CAC1996** | Hypothetical protein | 0.2 | 2.62±0.9 | 0.53±0.01 |
| **CAC1997** | Predicted glycosyltransferase | 0.21 | 2.72±1.04 | 0.56±0.02 |
| **CAC1998** | ABC-type transport system, ATPase component | 0.19 | 2.42±0.94 | 0.46±0.03 |
| **CAC1999** | Uncharacterized protein related to hypothetical protein Cj1507c from Campylobacter jejuni | 0.19 | 2.15±0.9 | 0.4±0.02 |
| **CAC2000** | Indolepyruvate ferredoxin oxidoreductase, subunit beta | 0.17 | 2.65±1.09 | 0.44±0.06 |
| **CAC2001** | Indolepyruvate ferredoxin oxidoreductase, subunit alpha | 0.22 | 9.05±4.28 | 2±0.15 |
| **CAC2002** | Predicted iron-sulfur flavoprotein | 0.24 | 3.57±1.27 | 0.85±0.02 |
| **CAC2003** | Predicted permease | 0.21 | 1.7±0.84 | 0.36±0.03 |
| **CAC2004** | Siderophore/Surfactin synthetase related protein | 0.21 | 6.96±2.59 | 1.43±0.02 |
| **CAC2005** | Siderophore/Surfactin synthetase related protein | 0.19 | 4.06±1.57 | 0.76±0.02 |
| **CAC2006** | Enzyme of siderophore/surfactin biosynthesis | 0.22 | 1.65±0.59 | 0.37±0.01 |
| **CAC2007** | Predicted glycosyltransferase | 0.21 | 8.79±3.64 | 1.85±0.09 |
| **CAC2009** | 3-Hydroxyacyl-CoA dehydrogenase | 0.22 | 6.35±1.95 | 1.42±0.06 |
| **CAC2010** | Predicted Fe-S oxidoreductase | 0.21 | 8.54±2.9 | 1.79±0.04 |
| **CAC2011** | Possible 3-oxoacyl-[acyl-carrier-protein] synthase III | 0.2 | 5.89±1.94 | 1.19±0.06 |
| **CAC2012** | Enoyl-CoA hydratase | 0.22 | 3.36±0.33 | 0.75±0.03 |
| **CAC2013** | Hypothetical protein | 0.19 | 8.36±2.44 | 1.56±0.02 |
| **CAC2014** | Predicted esterase | 0.19 | 8.21±2.59 | 1.58±0 |
| **CAC2015** | Hypothetical protein | 0.19 | 3.84±1.03 | 0.71±0.01 |
| **CAC2016** | Enoyl-CoA hydratase | 0.21 | 23.03±4.11 | 4.8±0.11 |
| **CAC2017** | Acyl carrier protein | 0.23 | 5.75±1.05 | 1.34±0.08 |
| **CAC2018** | Aldehyde:ferredoxin oxidoreductase | 0.18 | 6.52±2.32 | 1.16±0.14 |
| **CAC2019** | Malonyl CoA-acyl carrier protein transacylase | 0.19 | 6.63±2.11 | 1.29±0.03 |
| **CAC2020** | Molybdopterin biosynthesis enzyme, MoeA, fused to molibdopterin-binding domain | 0.15 | 0.85±0.32 | 0.12±0 |
| **CAC2021** | Molybdopterin biosynthesis enzyme, MoeA (short form) | 0.12 | 2.54±1.13 | 0.29±0.02 |
| **CAC2252** | Alpha-glucosidase fused to unknown alpha-amylase C-terminal. domain | 0.01 | 41.27±28.23 | 0.34±0.06 |
| **CAC2289** | Biotin carboxyl carrier protein | 0.2 | 0.45±0.06 | 0.09±0 |
| **CAC2514** | Beta galactosidase | 0.25 | 0.4±0.16 | 0.1±0 |
| **CAC2570** | Predicted arabinogalactan endo-1,4-beta-galactosidase | 0.09 | 4.75±1.31 | 0.43±0.02 |
| **CAC2610** | L-fucose isomerase related protein | 0.08 | 2.43±2.19 | 0.19±0 |
| **CAC2611** | Hypothetical protein | 0.07 | 2.57±2.57 | 0.19±0.01 |
| **CAC2774** | Methyl-accepting chemotaxis protein with HAMP domain | 0.14 | 3.25±1.35 | 0.45±0.03 |
| **CAC2805** | Possible selenocysteine lyase (aminotransferase of NifS family) | 0.21 | 0.39±0.14 | 0.08±0.01 |
| **CAC2806** | Predicted phosphohydrolase, Icc family | 0.1 | 79.67±1.72 | 7.74±1.19 |
| **CAC2807** | Endo-1,3(4)-beta-glucanase family 16 | 0.04 | 64.7±11.05 | 2.77±1.21 |
| **CAC2808** | Beta-lactamase class C domain (PBPX family) containing protein | 0.16 | 1.79±0.66 | 0.29±0.01 |
| **CAC2809** | Predicted HD superfamily hydrolase | 0 | 1.45±1.12 | 0±0 |
| **CAC2810** | Possible glucoamylase (diverged), 15 family | 0.03 | 3.81±0.79 | 0.13±0.01 |
| **CAC2833** | Uncharacterized conserved protein, YAEG family | 0.06 | 1.14±0.4 | 0.07±0 |
| **CAC2834** | Uncharacterized conserved protein, YHAD family | 0.01 | 40.02±7.86 | 0.2±0.01 |
| **CAC2835** | Gluconate permease, gntP | 0 | 35.73±17.74 | 0.12±0.01 |
| **CAC2847** | Ribosome-associated protein Y (PSrp-1) | 0.23 | 14.71±4.59 | 3.34±0.15 |
| **CAC2891** | Fusion of alpha-glucosidase (family 31 glycosyl hydrolase) and glycosidase (TreA/MalS family) | 0.07 | 6.1±4.66 | 0.43±0.01 |
| **CAC2959** | Galactokinase | 0.09 | 8.67±3.42 | 0.76±0.05 |
| **CAC2960** | UDP-galactose 4-epimerase | 0.1 | 2.42±0.92 | 0.23±0.01 |
| **CAC2961** | Galactose-1-phosphate uridyltransferase | 0.14 | 2.81±0.83 | 0.4±0.02 |
| **CAC2962** | Transcriptional regulators of the LacI family | 0.19 | 5.49±2.78 | 1.07±0.06 |
| **CAC3032** | Galactose mutarotase related enzyme | 0.16 | 4.88±0.1 | 0.79±0.01 |
| **CAC3157** | Tryptophan synthase alpha chain | 0.25 | 3.02±2.11 | 0.75±0.08 |
| **CAC3158** | Tryptophan synthase beta chain | 0.19 | 14.1±10.61 | 2.67±0.26 |
| **CAC3159** | Phosphoribosylanthranilate isomerase | 0.12 | 9.4±6.76 | 1.12±0.12 |
| **CAC3160** | Indole-3-glycerol phosphate synthase | 0.12 | 5.36±4.12 | 0.63±0.06 |
| **CAC3161** | Anthranilate phosphoribosyltransferase | 0.12 | 4.06±2.91 | 0.47±0.03 |
| **CAC3162** | Para-aminobenzoate synthase component II | 0.08 | 6.09±4.75 | 0.47±0.02 |
| **CAC3163** | Para-aminobenzoate synthase component I | 0.09 | 1.64±1.18 | 0.14±0.01 |
| **CAC3236** | Possible transcriptional regulator from YAEG/LRPR family | 0.2 | 2.26±1.1 | 0.45±0.01 |
| **CAC3237** | Multiple sugar-binding ABC-transporter, MSMX ATP-binding protein | 0.23 | 1.42±0.56 | 0.33±0.02 |
| **CAC3362** | Uncharacterized conserved membrane protein, YOAK B.subtilis homolog | 0.21 | 0.62±0.05 | 0.13±0.02 |
| **CAC3425** | PTS system, (possibly glucose-specific) IIBC component | 0 | 0.23±0.12 | 0±0 |
| **CAC3489** | Hypothetical protein | 0.23 | 1.13±0.11 | 0.26±0.01 |
| **CAC3498** | Sugar kinase, ribokinase family | 0.19 | 0.46±0.16 | 0.09±0.02 |
| **CAC3612** | Hypothetical protein | 0.09 | 3.49±1.51 | 0.33±0.02 |
| **CAC3613** | Hypothetical protein | 0.18 | 0.83±0.5 | 0.15±0.01 |
| **CAC3617** | Uncharacterized membrane protein, YHAG B.subtilis homolog | 0.13 | 0.73±0.48 | 0.1±0 |
| **CAC3671** | ABC-type sugar transport system, permease component | 0 | 0.24±0.11 | 0±0 |
| **CAC3672** | ABC-type sugar transport system, periplasmic sugar-binding component | 0.2 | 0.31±0.13 | 0.06±0 |
| **CAP0066** | Mannose-specific phosphotransferase system component IIAB | 0.08 | 15.39±2.91 | 1.24±0.13 |
| **CAP0067** | Mannose/fructose-specific phosphotransferase system component IIC | 0.08 | 29.27±6.73 | 2.24±0.13 |
| **CAP0068** | Mannose-specific phosphotransferase system component IID | 0.06 | 17.54±3.27 | 1.04±0.03 |
| **CAP0069** | Uncharacterized protein, homolog of Streptococcus salivarius (5669858) | 0.08 | 5.56±2.78 | 0.45±0.09 |
| **CAP0072** | Hypothetical protein | 0.13 | 2.68±0.98 | 0.36±0.05 |
| **CAP0098** | Alpha-amylase, AmyB | 0.17 | 0.44±0.13 | 0.07±0.02 |
| **CAP0162** | NAD+ dependent aldehyde dehydrogenase (adhE1) | 0 | 7.09±0.73 | 0±0 |

**Table S5. Four-fold increased or decreased genes under solventogenesis in *ΔadhE2***

| **Gene number** | **Function** | **adhE2**  **/Ctrl** | **Control** | **adhE2** |
| --- | --- | --- | --- | --- |
| **Increase** |  |  |  |  |
| **CAC1043** | Xre family DNA-binding domain and TPR-repeat containing protein | 4.13 | 0.1±0.01 | 0.43±0.23 |
| **CAC1880** | Hypothetical protein, CF-35 family | 4.29 | 0.12±0.01 | 0.52±0.32 |
| **CAC1881** | Hypothetical protein | 4.83 | 0.11±0.01 | 0.55±0.36 |
| **CAC1885** | Hypothetical protein | ∞ | 0±0 | 0.32±0.21 |
| **CAC1886** | Uncharacterized phage related protein | ∞ | 0±0 | 0.42±0.28 |
| **CAC1887** | Hypothetical protein | ∞ | 0±0 | 0.38±0.26 |
| **CAC1888** | Uncharacterized phage related protein | ∞ | 0±0 | 1.08±0.82 |
| **CAC1892** | Hypothetical protein | ∞ | 0±0 | 0.32±0.21 |
| **CAC1893** | ClpP family serine protease, possible phage related | ∞ | 0±0 | 1.41±1.07 |
| **CAC1894** | Phage-related, head portal protein | ∞ | 0±0 | 0.29±0.19 |
| **CAC1897** | Phage-related, Zn finger domain containing protein | ∞ | 0±0 | 0.29±0.19 |
| **CAC1945** | Phage related anti-repressor protein | ∞ | 0±0 | 0.22±0.13 |
| **CAC2438** | Predicted phosphatase | 4.26 | 0.17±0.09 | 0.73±0.81 |
| **CAC3234** | Uncharacterized conserved protein, YVBJ B.subtilis ortholog with N-terminal C4-type Zn-finger domain | 4.79 | 0.23±0.06 | 1.11±0.9 |
| **CAC3236** | Possible transcriptional regulator from YAEG/LRPR family | 8.76 | 2.26±1.1 | 19.75±17.13 |
| **CAC3237** | Multiple sugar-binding ABC-transporter, MSMX ATP-binding protein | 7.44 | 1.42±0.56 | 10.6±8.4 |
| **CAC3379** | Uncharacterized protein, YQFW B.subtilis homolog | 4.37 | 0.78±0.57 | 3.41±3.85 |
| **CAC3604** | Dihydroxyacid dehydratase | 122.68 | 0.18±0.04 | 22.11±12.55 |
| **CAC3605** | High affinity gluconate/L-idonate permease | 127.91 | 0.13±0.03 | 16.54±10.3 |
| **CAP0029** | Permease MDR-related | 20.5 | 0.14±0.09 | 2.88±0.72 |
| **CAP0030** | Isochorismatase | 22.96 | 0.23±0.15 | 5.29±1.7 |
| **CAP0031** | Transcriptional activator HLYU, HTH of ArsR family | 9.57 | 0.69±0.15 | 6.62±2.03 |
|  |  |  |  |  |
| **Decrease** |  |  |  |  |
| **CAC0014** | Aminotransferase | 0.09 | 3.73±1.51 | 0.34±0.03 |
| **CAC0015** | D-3-phosphoglycerate dehydrogenase | 0.08 | 6.29±2.56 | 0.51±0.08 |
| **CAC0016** | Related to HTH domain of SpoOJ/ParA/ParB/repB family, involved in chromosome partitioning | 0.14 | 1.66±1.48 | 0.23±0.01 |
| **CAC0017** | Seryl-tRNA synthetase | 0.11 | 1.04±0.51 | 0.12±0.01 |
| **CAC0078** | Accessory gene regulator protein B | 0 | 1.99±0.03 | 0±0 |
| **CAC0079** | Hypothetical protein | 0 | 68.44±1.59 | 0.1±0.03 |
| **CAC0082** | Predicted membrane protein | 0.02 | 42.74±3.17 | 0.81±0.4 |
| **CAC1634** | Flagellin | 0.25 | 2.42±1.98 | 0.6±0.25 |
| **CAC2569** | NimC/NimA family protein | 0.22 | 7.73±3.94 | 1.71±0.39 |
| **CAC3408** | NADH oxidase (two distinct flavin oxidoreductase domains) | 0.04 | 6.6±0.71 | 0.28±0.13 |
| **CAC3409** | Transcriptional regulators, LysR family | 0.02 | 20.17±3.06 | 0.48±0.33 |
| **CAC3422** | Sugar:proton symporter (possible xylulose) | 0.02 | 9.01±2.14 | 0.16±0.07 |
| **CAC3423** | Acetyltransferase (ribosomal protein N-acetylase subfamily) | 0.03 | 10.41±1.56 | 0.28±0.06 |
| **CAC3424** | Transcriptional regulator, RpiR family | 0.23 | 1.71±0.18 | 0.39±0.07 |
| **CAC3612** | Hypothetical protein | 0.22 | 3.49±1.51 | 0.75±0.45 |
| **CAP0028** | HTH transcriptional regulator TetR family | 0.18 | 0.45±0.05 | 0.08±0 |
| **CAP0035** | NADH-dependent aldehyde/alcohol dehydrogenase (adhE2) | 0 | 0.21±0.02 | 0±0 |

**Table S6. Four-fold increased or decreased genes under alcohologenesis in *ΔadhE1***

| **Gene number** | **Function** | **adhE2**  **/Ctrl** | **Control** | **adhE2** |
| --- | --- | --- | --- | --- |
| **Increase** |  |  |  |  |
| **CAC0078** | Accessory gene regulator protein B | 4.32 | 0.54±0.2 | 2.32±0.13 |
|  |  |  |  |  |
| **Decrease** |  |  |  |  |
| **CAC0422** | Transcriptional antiterminator licT | 0.11 | 2.55±1.5 | 0.27±0.02 |
| **CAC0423** | Fusion: PTS system, beta-glucosides specific IIABC component | 0.01 | 14.55±9.31 | 0.1±0.01 |
| **CAC0424** | Fructokinase | 0.02 | 5.66±3.62 | 0.09±0.01 |
| **CAC0425** | Sucrase-6-phosphate hydrolase (gene sacA) | 0.02 | 3.33±2.1 | 0.07±0 |
| **CAC0426** | Transcriptional regulator (HTH_ARAC-domain) | 0.13 | 47.23±26.81 | 5.92±0.61 |
| **CAC0751** | Permease | 0.17 | 3.83±0.22 | 0.64±0.04 |
| **CAC1406** | Transcriptional antiterminator (BglG family) | 0.14 | 25.57±13.31 | 3.45±0.42 |
| **CAC1407** | PTS system, beta-glucosides-specific IIABC component | 0.12 | 0.9±0.51 | 0.11±0.01 |
| **CAC1408** | Phospho-beta-glucosidase | 0.12 | 1.23±0.68 | 0.15±0.01 |
| **CAC3274** | Possible surface protein, responsible for cell interaction; contains cell adhesion domain and ChW-repeats | 0.17 | 1.43±0.94 | 0.24±0.02 |
| **CAC3459** | Homolog of cell division GTPase FtsZ, diverged | 0.23 | 1.79±1.12 | 0.41±0.02 |
| **CAP0029** | Permease MDR-related | 0 | 0.81±0.53 | 0±0 |
| **CAP0030** | Isochorismatase | 0.04 | 1.84±1.26 | 0.08±0 |
| **CAP0031** | Transcriptional activator HLYU, HTH of ArsR family | 0.25 | 2.5±1.37 | 0.62±0.07 |

**Table S7**. Four-fold increased or decreased genes under alcohologenesis in *ΔadhE2*

| **Gene number** | **Function** | **adhE2**  **/Ctrl** | **Control** | **adhE2** |
| --- | --- | --- | --- | --- |
| **Increase** |  |  |  |  |
| **CAC0265** | Transcriptional regulator, GntR family | 4.39 | 0.91±0.05 | 4±0.08 |
| **CAC0266** | ABC transporter, ATP-binding protein | 4.97 | 0.41±0.06 | 2.05±0.06 |
| **CAC0375** | PLP-dependent aminotransferase (gene patA) | 4.1 | 3.77±0.32 | 15.45±0.98 |
| **CAC0682** | Ammonium transporter (membrane protein nrgA) | 11.43 | 0.31±0.05 | 3.53±0.19 |
| **CAC1107** | Hypothetical protein, CF-36 family | 4.52 | 0.08±0.02 | 0.37±0.02 |
| **CAC1130** | Hypothetical protein | 4.78 | 0.08±0.02 | 0.39±0 |
| **CAC1131** | Hypothetical protein | 4.91 | 0.08±0.02 | 0.41±0.01 |
| **CAC1600** | Methyl-accepting chemotaxis-like protein (chemotaxis sensory transducer) | 4.95 | 0.99±0.6 | 4.88±0.16 |
| **CAC1601** | Methyl-accepting chemotaxis-like protein (chemotaxis sensory transducer) | 5.64 | 0.58±0.3 | 3.3±0.16 |
| **CAC1634** | Flagellin | 4.53 | 1.99±1.39 | 9.03±1.02 |
| **CAC1705** | Periplasmic phosphate-binding protein | 27.3 | 0.27±0.09 | 7.33±0.53 |
| **CAC1706** | Phosphate permease | 6.66 | 0.08±0.03 | 0.56±0.09 |
| **CAC1707** | Permease component of ATP-dependent phosphate uptake system | 11.24 | 0.09±0.01 | 1.05±0.1 |
| **CAC1708** | ATPase component of ABC-type phosphate transport system | 20.22 | 0.13±0.01 | 2.64±0.15 |
| **CAC1709** | Phosphate uptake regulator | 5.83 | 0.12±0.02 | 0.69±0.03 |
| **CAC2203** | Possible hook-associated protein, flagellin family | 4.42 | 12.36±8.44 | 54.69±3.54 |
| **CAC2717** | Ethanolamine ammonia lyase small subunit | 4.1 | 0.12±0 | 0.48±0.02 |
| **CAC2718** | Ethanolamine ammonia lyase large subunit | 5.11 | 0.12±0.01 | 0.59±0.03 |
| **CAC2746** | Membrane associated methyl-accepting chemotaxis protein (with HAMP domain) | 6.76 | 0.22±0.12 | 1.49±0.03 |
| **CAC3352** | Membrane associated methyl-accepting chemotaxis protein with HAMP domain | 4.79 | 0.62±0.32 | 2.96±0.19 |
| **CAC3604** | Dihydroxyacid dehydratase | 297.03 | 0.19±0.03 | 57.09±1 |
| **CAC3605** | High affinity gluconate/L-idonate permease | 301.06 | 0.13±0.01 | 38.52±4.31 |
| **CAP0029** | Permease MDR-related | 11.43 | 0.81±0.53 | 9.28±0.99 |
| **CAP0030** | Isochorismatase | 14.44 | 1.84±1.26 | 26.58±0.98 |
| **CAP0031** | Transcriptional activator HLYU, HTH of ArsR family | 10.71 | 2.5±1.37 | 26.77±2.14 |
| **CAP0036** | Uncharacterized, ortholog of YgaT gene of B.subtillis | 9.55 | 1.97±0.18 | 18.8±1.04 |
| **CAP0037** | Uncharacterized, ortholog of YgaS gene of B.subtillis | 8.94 | 1.61±0.11 | 14.43±0.9 |
| **CAP0045** | Glycosyl transferase | 5.06 | 0.64±0.08 | 3.25±0.15 |
| **CAP0087** | HTH transcriptional regulator TetR/AcrR family | 13.65 | 0.84±0.09 | 11.53±0.47 |
| **CAP0088** | 3-oxoacyl-acyl-carrier protein synthase | 21.46 | 2.45±0.23 | 52.58±2.19 |
| **CAP0167** | Specialized sigma factor (SigF/SigE family) | 5.32 | 0.42±0.25 | 2.22±0.22 |
| **CAP0169** | Hypothetical protein, CF-45 family | 5.77 | 0.15±0.07 | 0.87±0.15 |
| **CAP0170** | Hypothetical protein, CF-46 family | 8.35 | 0.08±0.01 | 0.69±0.13 |
| **CAP0171** | Hypothetical protein, CF-45 family | 7.01 | 0.1±0.02 | 0.67±0.15 |
| **CAP0172** | Hypothetical protein, CF-46 family | 6.71 | 0.4±0.11 | 2.69±0.77 |
|  |  |  |  |  |
| **Decrease** |  |  |  |  |
| **CAC0078** | Accessory gene regulator protein B | 0 | 0.54±0.2 | 0±0 |
| **CAC0079** | Hypothetical protein | 0 | 10.91±8.15 | 0±0 |
| **CAC0082** | Predicted membrane protein | 0.03 | 15.51±5.65 | 0.43±0.03 |
| **CAC0316** | Ornithine carbomoyltransferase | 0.11 | 6.54±1.37 | 0.75±0.03 |
| **CAC0380** | Periplasmic amino acid-binding protein | 0.21 | 7.74±1.74 | 1.63±0.08 |
| **CAC0706** | Endo-1,4-beta glucanase (fused to two ricin-B-like domains) | 0.14 | 5.11±2.59 | 0.69±0.08 |
| **CAC0973** | Argininosuccinate synthase | 0.11 | 10.23±0.45 | 1.15±0.09 |
| **CAC0974** | Argininosuccinate lyase | 0.1 | 11.11±0.44 | 1.11±0 |
| **CAC1319** | Glycerol uptake facilitator protein, GLPF | 0.23 | 35.83±8.36 | 8.34±1.13 |
| **CAC1320** | Glycerol-3-phosphate responsive antiterminator (mRNA-binding), GLPP | 0.2 | 16.28±3.56 | 3.26±0.51 |
| **CAC1321** | Glycerol kinase, GLPK | 0.21 | 27.85±6.42 | 5.83±0.61 |
| **CAC1322** | Glycerol-3-phosphate dehydrogenase, GLPA | 0.18 | 59.33±8.11 | 10.51±1.61 |
| **CAC1323** | NAD(FAD)-dependent dehydrogenase | 0.23 | 58.91±8.35 | 13.49±1.21 |
| **CAC1324** | Uncharacterized predected metal-binding protein | 0.22 | 40.66±1.55 | 8.97±0.95 |
| **CAC1405** | Beta-glucosidase | 0.11 | 16.94±4.45 | 1.78±0.11 |
| **CAC1888** | Uncharacterized phage related protein | 0.19 | 0.48±0.15 | 0.09±0 |
| **CAC1893** | ClpP family serine protease, possible phage related | 0.19 | 0.56±0.14 | 0.1±0.01 |
| **CAC2388** | N-acetylornithine aminotransferase | 0.1 | 6.84±0.09 | 0.68±0.04 |
| **CAC2389** | Acetylglutamate kinase | 0.24 | 0.48±0.12 | 0.11±0.01 |
| **CAC2390** | N-acetyl-gamma-glutamyl-phosphate reductase | 0.17 | 1.55±0.26 | 0.26±0.01 |
| **CAC2391** | Ornithine acetyltransferase | 0.2 | 2.86±0.63 | 0.57±0.02 |
| **CAC2456** | Hypothetical protein, CF-40 family | 0.08 | 3.72±1.92 | 0.31±0.03 |
| **CAC2457** | Hypothetical protein | 0.1 | 3.66±1.91 | 0.38±0.01 |
| **CAC2469** | Lactoylglutathione lyase (fragment) | 0.23 | 0.92±0.24 | 0.21±0.02 |
| **CAC2470** | Uncharacterized Zn-finger protein | 0.25 | 1.94±0.4 | 0.48±0.03 |
| **CAC2511** | Predicted membrane protein | 0.24 | 0.46±0.22 | 0.11±0 |
| **CAC2644** | Carbamoylphosphate synthase large subunit | 0.22 | 2.31±0.23 | 0.51±0.08 |
| **CAC2645** | Carbamoylphosphate synthase small subunit | 0.23 | 0.53±0.12 | 0.12±0.02 |
| **CAC3160** | Indole-3-glycerol phosphate synthase | 0.24 | 2.58±0.63 | 0.63±0.06 |
| **CAC3274** | Possible surface protein, responsible for cell interaction; contains cell adhesion domain and ChW-repeats | 0.15 | 1.43±0.94 | 0.21±0.03 |
| **CAC3408** | NADH oxidase (two distinct flavin oxidoreductase domains) | 0.04 | 3.28±1.4 | 0.12±0 |
| **CAC3409** | Transcriptional regulators, LysR family | 0.02 | 9.93±2.4 | 0.15±0.01 |
| **CAC3422** | Sugar:proton symporter (possible xylulose) | 0.07 | 2.71±0.43 | 0.19±0.01 |
| **CAC3423** | Acetyltransferase (ribosomal protein N-acetylase subfamily) | 0.08 | 3.19±0.7 | 0.27±0.01 |
| **CAC3486** | Multimeric flavodoxin WrbA family protein | 0.23 | 2.28±1.03 | 0.52±0.03 |
| **CAC3618** | ABC-type polar amino acid transport system, ATPase component | 0.22 | 4.89±0.41 | 1.09±0.02 |
| **CAP0028** | HTH transcriptional regulator TetR family | 0.24 | 0.53±0.07 | 0.13±0.01 |
| **CAP0035** | NADH-dependent aldehyde/alcohol dehydrogenase (adhE2) | 0 | 68.6±12.95 | 0±0 |

**A**


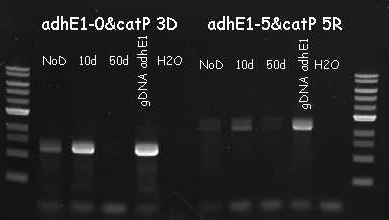

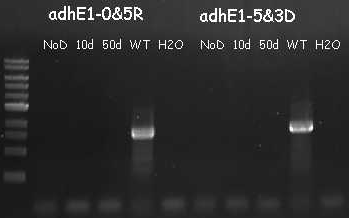


**B**


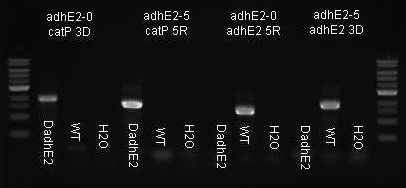


**Fig. S1. PCR Verification of deletion of *adhE1* in *ΔadhE1* strain (A) and *adhE2* in *ΔadhE2* strain (B).** adhEX-0 is 5’ external primer. adhEX-5 is 3’ external primer. adhEX-5R/3D are located on target gene. catP 5R/3D are located on catP cassette. Abbreviations used in this figure: Nod, Non diluted culture; 10d, 10 times diluted culture; 50d, 50 times diluted culture; WT, genomic DNA of *C. acetobutylicum* ATCC 824; gDNA *adhE1*, genomic DNA of *ΔadhE1*; DadhE2. Genomic DNA of *ΔadhE2.*

***Acidogenesis***

**Fig. S2.** **Butanol pathway analysis of control (A), *ΔadhE1* (B)*, ΔadhE2* (C) under acidogenesis, solventogenesis, and alcohologenesis.** Activity distributions of the five enzymes are shown for each step under the arrows. The primary cofactors used for each step are shown over the arrows. Butanol flux is indicated under the word “Butanol.”

***Solventogenesis***

**Fig. S2. Butanol pathway analysis of control (A), *ΔadhE1* (B)*, ΔadhE2* (C) under acidogenesis, solventogenesis, and alcohologenesis.** Activity distributions of the five enzymes are shown for each step under the arrows. The primary cofactors used for each step are shown over the arrows. Butanol flux is indicated under the word “Butanol.”

***Alcohologenesis***

**Fig. S2.** **Butanol pathway analysis of control (A), *ΔadhE1* (B)*, ΔadhE2* (C) strains under acidogenesis, solventogenesis, and alcohologenesis.** Activity distributions of the five enzymes are shown for each step under the arrows. The primary cofactors used for each step are shown over the arrows. Butanol flux is indicated under the word “Butanol.”

**Fig. S3. Metabolic flux map of *ΔadhE1* under acidogenesis (A), *ΔadhE2* under acidogenesis (B), *ΔadhE1* under solventogenesis (C), *ΔadhE2* under solventogenesis (D), . *ΔadhE1* under alcohologenesis (E), *ΔadhE2* under alcohologenesis (F).** All values are normalized to the flux of the initial carbon source (mmol/gDCW/h). Glucose flux is normalized as 100 for acidogenesisand solventogenesis, and the sum of glucose and half of the glycerol normalized as 100 for alcohologenesis. The values of corresponding mutant are shown in blue letter, and that of control strain are shown in green letter.

**Fig. S3. Metabolic flux map of *ΔadhE1* under acidogenesis (A), *ΔadhE2* under acidogenesis (B), *ΔadhE1* under solventogenesis (C), *ΔadhE2* under solventogenesis (D), . *ΔadhE1* under alcohologenesis (E), *ΔadhE2* under alcohologenesis (F)**. All values are normalized to the flux of the initial carbon source (mmol/gDCW/h). Glucose flux is normalized as 100 for acidogenesisand solventogenesis, and the sum of glucose and half of the glycerol normalized as 100 for alcohologenesis. The values of corresponding mutant are shown in blue letter, and that of control strain are shown in green letter.

**Fig. S3. Metabolic flux map of *ΔadhE1* under acidogenesis (A), *ΔadhE2* under acidogenesis (B), *ΔadhE1* under solventogenesis (C), *ΔadhE2* under solventogenesis (D), . *ΔadhE1* under alcohologenesis (E), *ΔadhE2* under alcohologenesis (F)**. All values are normalized to the flux of the initial carbon source (mmol/gDCW/h). Glucose flux is normalized as 100 for acidogenesisand solventogenesis, and the sum of glucose and half of the glycerol normalized as 100 for alcohologenesis. The values of corresponding mutant are shown in blue letter, and that of control strain are shown in green letter.

**Fig. S3. Metabolic flux map of *ΔadhE1* under acidogenesis (A), *ΔadhE2* under acidogenesis (B), *ΔadhE1* under solventogenesis (C), *ΔadhE2* under solventogenesis (D), . *ΔadhE1* under alcohologenesis (E), *ΔadhE2* under alcohologenesis (F)**. All values are normalized to the flux of the initial carbon source (mmol/gDCW/h). Glucose flux is normalized as 100 for acidogenesisand solventogenesis, and the sum of glucose and half of the glycerol normalized as 100 for alcohologenesis. The values of corresponding mutant are shown in blue letter, and that of control strain are shown in green letter.

**Fig. S3. Metabolic flux map of *ΔadhE1* under acidogenesis (A), *ΔadhE2* under acidogenesis (B), *ΔadhE1* under solventogenesis (C), *ΔadhE2* under solventogenesis (D), . *ΔadhE1* under alcohologenesis (E), *ΔadhE2* under alcohologenesis (F)**. All values are normalized to the flux of the initial carbon source (mmol/gDCW/h). Glucose flux is normalized as 100 for acidogenesisand solventogenesis, and the sum of glucose and half of the glycerol normalized as 100 for alcohologenesis. The values of corresponding mutant are shown in blue letter, and that of control strain are shown in green letter.

**Fig. S3. Metabolic flux map of *ΔadhE1* under acidogenesis (A), *ΔadhE2* under acidogenesis (B), *ΔadhE1* under solventogenesis (C), *ΔadhE2* under solventogenesis (D), . *ΔadhE1* under alcohologenesis (E), *ΔadhE2* under alcohologenesis (F)**. All values are normalized to the flux of the initial carbon source (mmol/gDCW/h). Glucose flux is normalized as 100 for acidogenesisand solventogenesis, and the sum of glucose and half of the glycerol normalized as 100 for alcohologenesis. The values of corresponding mutant are shown in blue letter, and that of control strain are shown in green letter.

**References**

Croux, C., N.P.T. Nguyen, J. Lee, C. Raynaud, F. Saint-Prix, M. Gonzalez-Pajuelo, I. Meynial-Salles & P. Soucaille, (2016) Construction of a restriction-less, marker-less mutant useful for functional genomic and metabolic engineering of the biofuel producer Clostridium acetobutylicum. *Biotechnology for Biofuel* **9**: 21.

Sillers, R., A. Chow, B. Tracy & E.T. Papoutsakis, (2008) Metabolic engineering of the non-sporulating, non-solventogenic Clostridium acetobutylicum strain M5 to produce butanol without acetone demonstrate the robustness of the acid-formation pathways and the importance of the electron balance. *Metab Eng* **10**: 321-332.

Tummala, S.B., N.E. Welker & E.T. Papoutsakis, (1999) Development and characterization of a gene expression reporter system for Clostridium acetobutylicum ATCC 824. *Appl Environ Microbiol* **65**: 3793-3799.

Yoo, M., G. Bestel-Corre, C. Croux, A. Riviere, I. Meynial-Salles & P. Soucaille, (2015) A Quantitative System-Scale Characterization of the Metabolism of Clostridium acetobutylicum. *mBio* **6**: e01808-01815.

Zigha, A., (2013) A process for butanol production. In*.*: Google Patents, pp.
